# High-resolution analysis of cell-state transitions in yeast suggests widespread transcriptional tuning by alternative starts

**DOI:** 10.1186/s13059-020-02245-3

**Published:** 2021-01-14

**Authors:** Minghao Chia, Cai Li, Sueli Marques, Vicente Pelechano, Nicholas M. Luscombe, Folkert J. van Werven

**Affiliations:** 1 The Francis Crick Institute, London, UK; 2 Okinawa Institute of Science and Technology Graduate University, Okinawa, Onna 904-0495 Japan; 3 School of Life Sciences, Sun Yat-sen University, Guangzhou, China; 4 SciLifeLab, Department of Microbiology, Tumor and Cell Biology, Karolinska Institutet, Solna, Sweden; 5 Okinawa Institute of Science & Technology Graduate University, Okinawa, 904-0495 Japan; 6 UCL Genetics Institute, University College London, London, WC1E 6BT UK

## Abstract

**Background:**

The start and end sites of messenger RNAs (TSSs and TESs) are highly regulated, often in a cell-type-specific manner. Yet the contribution of transcript diversity in regulating gene expression remains largely elusive. We perform an integrative analysis of multiple highly synchronized cell-fate transitions and quantitative genomic techniques in *Saccharomyces cerevisiae* to identify regulatory functions associated with transcribing alternative isoforms.

**Results:**

Cell-fate transitions feature widespread elevated expression of alternative TSS and, to a lesser degree, TES usage. These dynamically regulated alternative TSSs are located mostly upstream of canonical TSSs, but also within gene bodies possibly encoding for protein isoforms. Increased upstream alternative TSS usage is linked to various effects on canonical TSS levels, which range from co-activation to repression. We identified two key features linked to these outcomes: an interplay between alternative and canonical promoter strengths, and distance between alternative and canonical TSSs. These two regulatory properties give a plausible explanation of how locally transcribed alternative TSSs control gene transcription. Additionally, we find that specific chromatin modifiers Set2, Set3, and FACT play an important role in mediating gene repression via alternative TSSs, further supporting that the act of upstream transcription drives the local changes in gene transcription.

**Conclusions:**

The integrative analysis of multiple cell-fate transitions suggests the presence of a regulatory control system of alternative TSSs that is important for dynamic tuning of gene expression. Our work provides a framework for understanding how TSS heterogeneity governs eukaryotic gene expression, particularly during cell-fate changes.

## Introduction

The ends of messenger RNAs (mRNAs) produced by RNA polymerase II (Pol II) are formed at the site where transcription is initiated, generating the transcript start site (TSS), and at the site where polyadenylation occurs, also known as the transcript end site (TES) [1, 2]. Where Pol II starts transcribing and where polyadenylation sites are selected is fundamental to how mRNAs are generated and how gene expression is regulated. It is therefore surprising that the choice of TSS and TES is highly heterogeneous with most genes expressing multiple transcript isoforms, thereby leading to a high degree of transcript diversity [3]. Despite all efforts to understand how alternative TSSs and TESs control gene expression and overall protein expression, the physiological importance for transcript heterogeneity remains largely elusive.

Transcript heterogeneity is hypothesized to play important roles in development, health, and disease [4]. For instance, throughout the *Drosophila* life cycle, more than 40% of developmentally expressed genes alter their TSS usage [5]. In mice and humans, the average gene has at least four alternative promoters, hence TSSs [6]. Stage-specific differences in alternative TSS expression were detected in more than 5000 genes during mouse cerebellar development [7]. A recent study showed that the choice of alternative promoters was correlated with patient survival usage across many cancers [8]. Besides TSS heterogeneity, many studies have also uncovered the importance of alternative TES selection in gene regulation. Developmental or cell-type-specific alternative polyadenylation events in *C. elegans* affect where and when genes are expressed [9–11]. Likewise, mutations associated with cancer promote the usage of new TESs leading to truncated mRNA isoforms and aberrant protein expression [12, 13]. Thus, alternative TSSs and TESs are a hallmark of development and disease.

Changes in the usage of TSSs or TESs can affect gene expression with various outcomes. Differential TSS/TES usage can either generate mRNAs with differing untranslated regions (UTRs), or more rarely, transcripts encoding truncated protein isoforms [3, 14]. In the former case, changes in the 5′ or 3′ UTR sequence can influence mRNA transcript stability, localization, and translation efficiency [15, 16]. Small open reading frames (ORFs) in 5′ extended leader sequences can titrate ribosomes away from productive translation of the full-length ORF impacting protein production [17–22].

Different studies have used budding yeast to profile and characterize the diversity of alternative transcripts [3, 23–25]. A median of 26 transcript isoforms per gene were observed during regular growth conditions [3]. Frequently, stress or nutrient source shifting induce changes in TSS and TES usage, thereby regulating gene expression to suit the needs of the cell [3, 26, 27]. There are many single locus studies showing how transcription from upstream alternative TSSs results in gene repression in *cis* via transcription-coupled chromatin changes [17, 18, 28, 29]. This process, known as transcriptional interference, is widespread in yeast, but also present in mammalian cells [28, 30–32]. Conversely, examples of upstream transcription activating downstream promoters have also been reported [33, 34]. Thus, TSS/TES usage changes can have varying outcomes on gene expression. However, the extent to which this mode of regulation occurs genome-wide is less well understood.

The budding yeast gametogenesis program, during which a diploid cell gives rise to four haploid spores, is an attractive model for studying the function of transcript heterogeneity on a genome-wide scale, because the program shares many features with a typical cell differentiation program. Like many developmental programs, during yeast gametogenesis, transcription is highly regulated by stage and DNA sequence-specific transcription factors [35–39]. The program can be divided into two synchronized cell-fate transitions, which can be controlled by an inducible expression system of two transcription factors, Ime1 and Ndt80 [40, 41]. Importantly, there is evidence of widespread expression of 5′ extended transcript isoforms that control protein expression in a cell-fate stage-specific manner during yeast gametogenesis [18, 19, 42]. Genome-wide examination of the cell-fate-specific changes in transcript isoform usage may reveal regulatory principles evoked by transcript heterogeneity, perhaps not observed under regular asynchronous growth conditions.

Here, we performed a multifaceted time-course analysis to identify regulatory principles linked to transcript isoform usage changes. Specifically, we examined transcript isoform usage levels during three highly synchronized cell-fate transitions that are part of the yeast gametogenesis program and the mitotic cell cycle. We found that the usage of alternative TSSs, and to lesser degree, TESs, changes dynamically during each cell-fate transition. Thousands of alternative TSS and TES clusters, upstream or downstream of the canonical TSSs, were upregulated in a stage-specific manner. Importantly, increased upstream alternative TSS usage was associated with a wide range of effects on canonical TSS usage levels, ranging from co-activation and co-expression to repression of canonical transcripts. We identified several regulatory features that explain various effects of alternative start usage on regulating gene expression. Our data suggest that TSS heterogeneity has a widespread function in tuning gene expression.

## Results

### Synchronizing three distinct cell-fate transitions in yeast

**Fig. 1:**
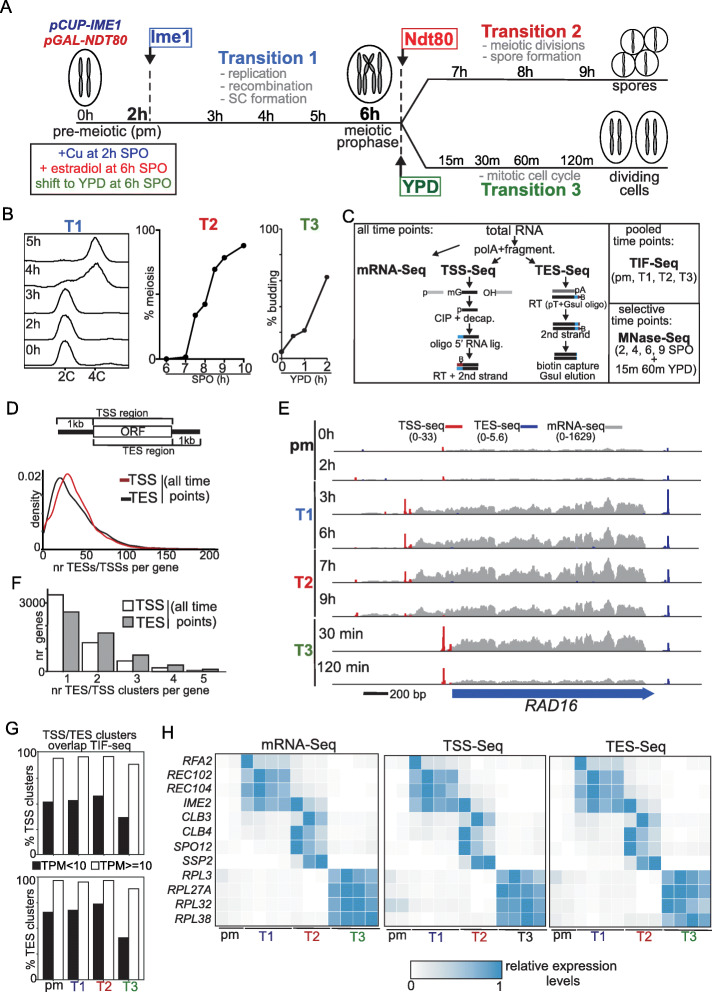
Profiling of transcript heterogeneity during three synchronized cell-fate transitions. **a** Schematic overview of the master time-course used for this study. Diploid cells harboring both *IME1* fused to the *CUP* promoter and *NDT80* expressed from the *GAL* promoter together with Gal4 fused to the estrogen receptor (*pCUP-IME1* and *GAL4.ER pGAL-NDT80*) (FW2795) were grown in rich medium (YPD) overnight. Saturated cultures were pelleted, washed, and resuspended (OD_600_ = 2.5) in sporulation medium (SPO). Samples were collected at the indicated time points, spanning the pre-meiotic (pm) cell-state and three different cell-fate transitions. Then, 50 μM CuSO_4_ was added 2 h after the cells were transferred to SPO to induce *IME1* expression and drive cells to enter meiosis (transition 1). Subsequently, 1 μM β-estradiol was added 6 h after transfer to SPO to induce *NDT80* expression, which in turn induced meiotic divisions and spore formation (transition 2). In parallel, at 6 h in SPO, cells were returned to the mitotic cell cycle (transition 3) by transferring cells to YPD. **b** Evidence for synchrony of cell-fate transition 1, 2, and 3. For transition 1 (T1), the kinetics of pre-meiotic DNA replication was determined by flow cytometry analysis of DNA content (left panel). Samples were taken at indicated time points and fixed, and DNA content was measured by propidium iodide staining. For transition 2 (T2), kinetics of meiotic divisions was determined. Samples were taken at the indicated time point and fixed in ethanol, nuclei were stained with DAPI, and DAPI masses were counted. Cells that harbored two, three, or four DAPI masses were classified as cells undergoing meiosis I or meiosis II (% meiosis). In total, 200 cells were counted at each time point. For transition 3, budding kinetics was determined by cell morphology (right panel) for 200 cells per time point. Results are representative of three independent, biological repeats. **c** Schematic of sample collections, TSS-seq and TES-seq methods and other methods were used. In short, we performed mRNA-seq after total RNA extraction. In addition, poly(A) + RNA was purified from aliquots of the same total RNA, was fragmented, and was used as inputs for TSS-seq or TES-seq. For TSS seq, the fragments were dephosphorylated and treated with a decapping enzyme so that only bona fide mRNA 5′ ends were competent for ligation. A custom oligo was ligated to these ends and fragments were converted to cDNA libraries for sequencing. For TES-seq, fragments harboring the 3′ ends were converted to cDNA using a biotinylated, anchored oligo d(T) primer with a GsuI restriction enzyme site. cDNA was then captured on streptavidin beads and the poly(A) tails were shortened by GsuI, before library amplification and sequencing. For TIF-seq, equal amounts of total RNA from each time point spanning the pre-meiotic stage (pm) and each cell-fate transition (T1–3) were pooled. For MNase-seq, cells at selected time points were harvested to profile chromatin structure. The data represented are from *n* = 3 biological repeats. **d** Distribution of the numbers of unique TSSs/TESs at single nucleotide resolution per gene. **e** Overview of mRNA-seq (gray), TSS seq (red), and TES seq (blue) data at the *RAD16* locus of different time points representing the different transitions (T1, T2, and T3). The scale of mRNA-seq, TSS-seq, and TES-seq values are depicted at the top of the panel. Scale (bp) are shown. **f** Distribution of the number of TSS/TES clusters per gene. **g** Percentage of TSS/TES clusters for each transition supported by TIF-seq. Weakly expressed TSSs/TESs (TPM < 10) are compared to the highly expressed ones (TPM ≥ 10). **h** Expression heatmap of genes known to be expressed early in gametogenesis (T1: *RFA2*, *REC102*, *REC104*, *IME2*), expressed after Ndt80 induction (T2: *CLB3, CLB4, SPO12, SSP2*) or expressed during mitotic growth (T3: *RPL3, RPL27a, RPL32, RPL38*). The pre-meiotic state (pm) is included as reference. mRNA-seq and TSS-seq and TES-seq data for each time point were scaled between 0 and 1 across the time course

To investigate how transcript diversity is regulated during cell-state transitions, we profiled different cell-fate transitions in yeast covering the gametogenesis program and re-entry into the mitotic cell cycle (Fig. 1a). A major benefit of the yeast model is that we can synchronize yeast gametogenesis and re-entry into the mitotic cell cycle, allowing for precise cell stage-specific measurements and minimizing effects caused by asynchronous cell populations. To obtain a high synchrony of three distinct cell fates, we used an engineered yeast strain that expressed the master regulatory transcription factor for entry into gametogenesis, *IME1*, from an inducible promoter (*pCUP-IME1*) [41]. The same strain also harbored the transcription factor *NDT80*, which controls meiotic divisions and spore formation, under control of a different inducible promoter (*pGAL-NDT80,* Gal4-ER) [40]. We designed a master time course with periodic sampling across three distinct cell-fate transitions: (Transition 1:) gametogenesis up until meiotic prophase by inducing Ime1 expression (*pCUP1-IME1*, +Cu), (Transition 2:) meiotic divisions followed by spore formation by inducing Ndt80 expression (*pGAl1-NDT80,* Gal4-ER, + β-estradiol), and (Transition 3:) re-entry into the mitotic cell division cycle (from meiotic prophase (6 h (h) in sporulation medium (SPO)) to nutrient-rich medium (YPD)). We defined these three cell-fate transitions as T1, T2, and T3, respectively (Fig. 1a).

To determine that cells underwent T1, T2, and T3 synchronously, we measured the synchrony of pre-meiotic DNA replication (T1), meiotic divisions (T2), and budding (T3) (Fig. 1b). Indeed, we found that most cells duplicated their DNA at 4 h in SPO (2 h after Ime1 induction), completed meiotic divisions at 9 h in SPO (2 h after Ndt80 induction), and displayed newly formed buds 2 h after shifting to rich nutrient conditions. These data confirm that our experimental system allows for synchronous progression through three distinct cell-state transitions.

### Quantitative profiling of transcript heterogeneity across multiple cell-fate transitions

We generated quantitative datasets of TSS and TES usage levels over multiple time points for each cell-fate transition (T1, T2, and T3). Specifically, we adopted high-throughput sequencing approaches to measure usage levels of TSSs (TSS-seq) and TESs (TES-seq) (Fig. 1c) [20, 43–45]. In addition, we measured mRNA expression at matching time points (mRNA-seq). To complement TSS-seq and TES-seq datasets, we also used an orthogonal method called transcript isoform sequencing (TIF-seq) on pooled samples of matching time points: prior to meiosis (pm), T1, T2, and T3 respectively [3]. The main utility of TIF-seq is that it matches the start and end of transcripts by sequencing the junction of circularized cDNAs spanning the 5′ and 3′ of transcripts, and can therefore precisely identify transcript isoforms [3, 46]. Finally, we determined nucleosome positions by micrococcal nuclease digestion of chromatin followed by high-throughput sequencing (MNase-seq) on selective time points across all three transitions [47]. The MNase-seq dataset allowed us to determine how the chromatin state is altered during each cell-fate transition. The combination of these methods provides a high-resolution view of transcript isoform diversity and chromatin states over multiple distinct cell-fate transitions (Fig. 1a, see materials and methods for how TSSs/TESs were filtered and assigned to genes).

At the single nucleotide level, we found that on average 38 TSSs and 39 TESs per gene were located within one kilobase (kb) upstream or downstream of the ORF in the sense orientation, which was in the range of what a previous study had showed [46] (Fig. 1d and Additional file 1: Fig. S1a). Individual TSSs and TESs often clustered together. For example, we detected about 256 TSS and 95 TES sites at the single nucleotide resolution at the *RAD16* locus, but most of them clustered within a few narrow regions (Fig. 1e). Therefore, we applied a computational method to define these TSS/TES clusters and identified 11,685 distinct TSS and 13,380 TES clusters respectively, with approximately half of the genes harboring two or more TSS (or TES) clusters (Fig. 1f and Additional file 1: Fig. S1b). Per time point, we identified between 7320 and 9412 TSS clusters and between 8437 and 11,382 TES clusters (Additional file 1: Fig. S1c). There was a good overlap between the TSS-seq/TES-seq and TIF-seq datasets (Additional file 1: Fig. S1e). At least 50% of TSS and TES clusters were detected in the TIF-seq dataset, even though TIF-seq was sequenced with lower read depth and displayed an over-representation of shorter fragments (Additional file: Fig. S1d). For the TSS/TES clusters with high expression (Tags Per Million reads (TPM) > = 10), more than 90% of them were supported by TIF-seq (Fig. 1g). Additionally, the three independent biological replicates in this study highly correlated with each other (Additional file 2). The TSS-seq and TES-seq datasets correlated well with the RNA-seq dataset (TSS-seq vs RNA-seq and TES-seq vs RNA-seq) (Fig. 1h and Additional file 1: Fig. S1f, S1g and Additional files 3, 4 and 5). However, it is worth noting that genes with relatively low expression levels for the RNA-seq correlated less well with TSS-seq and TES-seq, which could be due to noise in the data. Nevertheless, these data indicate that our TSS-seq and TES-seq datasets can be largely used for quantitative estimates of steady state levels of TSS and TES usage.

### Alternative TSSs and TESs are highly regulated by cell-fate-specific transcription factors

**Fig. 2:**
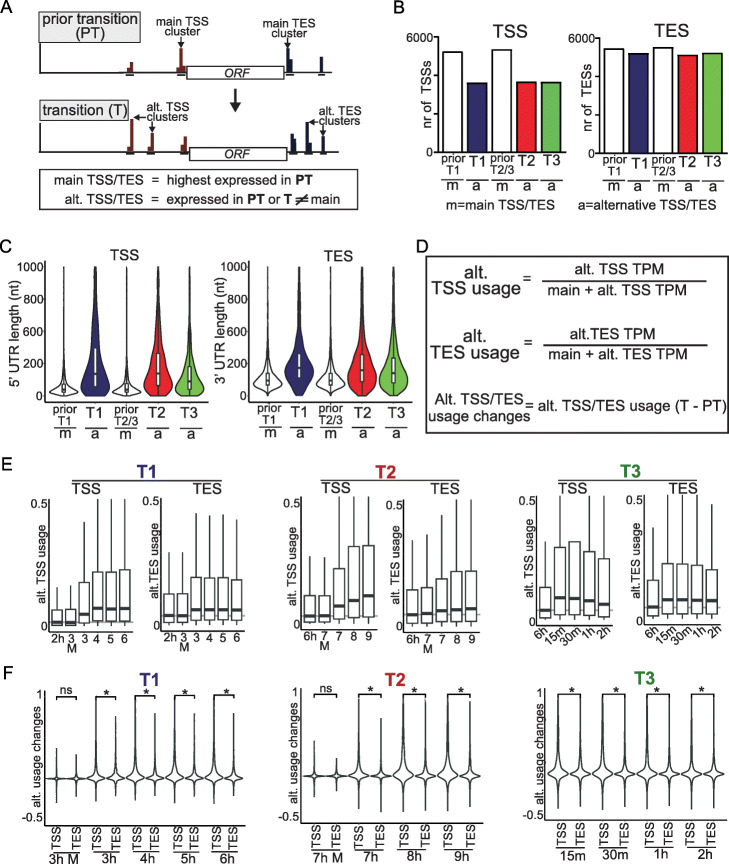
Alternative TSSs and TESs are pervasive expressed during T1, T2, and T3. **a** Schematic depicting the main and alternative TSSs (red) and TESs (blue) nearby a gene prior to and during cell-fate transitions (top). Definition of main and alternative TSS and TES (bottom). The main TSS/TES for T1 was defined as the most used TSS/TES in pre-meiotic cells (2 h). The main TSS/TES for T2 and 3 was defined as the most used TSS/TES during meiotic prophase (6 h). Any other TSS/TESs associated with the same gene were classed as alternative. TSS/TES clusters were only defined for a transition if they had a minimum level of expression (TPM ≥ 1) and were in the same orientation as the gene. **b** Number of distinct main (m) or alternative (a) TSS/TES clusters associated with genes for each cell-fate transition (T1, T2, and T3). **c** Distribution of 5′ (left) and 3′ (right) UTR lengths for main (m) or alternative (a) TSSs and TESs. The 5′ UTR length is defined as the distance given in number of nucleotides from the apex of a TSS cluster to the AUG of an annotated ORF. The 3′ UTR length is defined as the distance given in number of nucleotides from the apex of a TES cluster to the stop codon of an annotated ORF. Violin plots were scaled to a constant width. The alternative TSSs/TESs which were external to the ORF sequence and upregulated (two-fold or more) during T1 (*n* = 1118 for TSSs, and *n* = 1343 for TESs), T2 (*n* = 1079 for TSSs, and *n* = 1320 for TESs), or T3 (*n* = 1052 for TSSs, and *n* = 1611 for TESs) were used for this analysis. For the main TSSs/TESs *n* = 5016 and 5285 data points were used. The median position of main TSSs was 75 bp (T1) and 77 bp (T2 and T3), while that of the alternative TSSs upregulated during cell-fate transitions (2-fold or more) were at 170 bp (T1), 173 bp (T2) and 112 bp (T3) respectively. **d** Formulas for calculating alternative TSS/TES usage and alternative TSS/TES usage changes. Alternative TSS/TES usage for a gene was calculated by taking the alternative TSS/TES values divided over the sum of the main TSS/TES and alternative TSS/TES values. Alternative TSS/TES usage change was calculated by taking the difference in alternative TSS/TES usage between the transition (T) time point and the reference time point prior (PT) to transition. **e** Boxplots of alternative TSS and TES usage across different time points in T1, T2, and T3 using the formula defined in **d**. Negative controls (3 h, mock treated (3M) and 7 h, mock treated (7M)) representing cells which were shifted to SPO, but without inducing T1 or T2, were included. The alternative TSSs/TESs upregulated (two-fold or more) during T1, T2, or T3 were used for the analysis. **f** Similar to **e**, except that violin plots of changes in alternative TSS usage at different time points are displayed. Samples were compared using the Wilcoxon rank-sum test and * denotes *p* < 0.05

The terms *main* and *alternative* TSS and TES have been used in various ways. To avoid ambiguity, we defined these terms as the following. The *main* TSS or TES is the most highly expressed cluster prior to a cell-fate transition (PT, Fig. 2a). For T1, this was the most highly expressed TSS or TES cluster at the 2 h SPO time point, and for T2 or T3, the 6 h SPO time point. *Alternative* TSSs or TESs are the TSS or TES clusters expressed prior to or during cell-fate transitions, different from the main TSS/TES. Our definitions were fixed within each individual cell-fate transition (T1, T2, or T3).

Our analysis across three cell transitions revealed widespread usage of alternative TSS and TES clusters. For each cell-fate transition, we observed ~ 5800 alternative and main TESs, and ~ 3500 alternative and ~ 5800 main TSSs (Fig. 2b). Most alternative TSSs were expressed upstream of annotated ORFs, but a subset of genes harbored TSSs within the gene body (Additional file 1: Fig. S2, left panel). The median position of main TSSs was 75 bp (T1) and 77 bp (T2 and T3) upstream of the AUG of the matching ORF, while that of the alternative TSSs upregulated during cell-fate transitions (2 fold or more) were at 170 bp (T1), 173 bp (T2) and 112 bp (T3) respectively (Fig. 2c, Additional file 1: Fig. S2, right panel). A similar trend was observed for alternative TESs suggesting that increased 5′ and 3′ UTR lengths are characteristic of most alternative transcript isoforms expressed during cell-fate transitions.

Alternative TSSs and TESs were highly regulated across the three cell-fate transitions. Weighted gene correlation network analysis (WGCNA), which identifies the gene expression network based on expression correlation among genes across different time-points of the master time course, revealed 13 co-expression TSS modules and 15 TES modules, each consisting of at least one hundred genes (Additional file 1: Fig. S3a and S3b) [48]. The top three co-expression modules of TSSs and TESs were specifically upregulated during T1, T2, and T3 respectively (Additional file 1: Fig. S3a). Alternative TSSs and TESs were well represented in each expression module. In line with this observation, we found that transcription factors involved in regulating alternative and main TSSs were similar. The Ume6 binding motif was detected near main and alternative TSSs which were upregulated in T1, which is in line with the function of Ume6 together with Ime1 in activating transcription of the so-called “early” meiotic genes during yeast gametogenesis [49]. The binding motif of Ndt80, a transcription factor essential for activation of the middle and late meiotic genes, was enriched for T2 TSSs [50]. Given that expression of Ime1 and Ndt80 were controlled from heterologous promoters for T1 and T2 in order to obtain a highly synchronous cell population, there is a possibility that this can lead to mis-regulation of a subset transcripts. However, both synchronization methods have been used to study gene regulation during gametogenesis, and gave rise to viable spores that were indistinguishable from the wild-type [40, 41, 51]. Lastly, motifs of the transcriptional repressor Tod6 and transcriptional activator Sfp1 were proximal to the T3 TSSs (Additional file 1: Fig. S3c). Both transcription factors are known to regulate transcription of ribosomal protein gene promoters, and their activities are controlled by nutrient sensing kinases [52–54].

To test whether transcription factors directly control alternative TSS/TES usage, we compared TSS and TES changes between T1- and T2-induced cells (3H and 7H) and mock-treated cells of the matching time point (3M and 7M). We found that the vast majority of alternative TSSs and TESs were expressed in Ime1 and Ndt80-induced cells but not in the mock-treated cells for the same time period (3H versus 3M, and 7H versus 7M) (Additional file 1: Fig. S3d). We conclude that main and alternative TSSs/TESs are widely expressed through the action of cell-fate specific transcription factors.

### Increased main to alternative TSS usage is a common feature of cell-fate transitions

Next, we determined how alternative TSS and TES usage contributed to gene expression. Specifically, we computed the relative TSS and TES usage levels by taking the ratio of alternative versus the total TSSs/TESs levels of the same gene at the same time point (Fig. 2d). An increased ratio means elevated relative alternative TSS or TES usage, while a lower ratio indicates a decrease in relative usage. Proportional increases in expression from both alternative and main TSSs (e.g., if TSSs were not regulated independently) would result in an invariant ratio. Strikingly, we found that alternative TSS usage increased significantly during T1, T2, and T3 (Fig. 2d, e). For example, approximately 200–300 genes had alternative TSSs whose usage increased by at least 50% for T1, T2, and T3 respectively. In contrast, alternative TES usage changed by a smaller magnitude across the three transitions (Fig. 2e). Only 100–150 genes had alternative TESs whose usage increased 50% more than the main TES. The extent of increase of TSS was also significantly larger than TES (Fig. 2f, Wilcoxon rank sum test, *p* < 0.05). These increases were not seen for uninduced cells (Fig. 2e, see time points 3M and 7M). We conclude that there is a large shift from main to alternative TSS usage during cell-fate transitions. For remainder of the manuscript, we decided to focus on this remarkable observation.

### Increased upstream alternative TSS usage is linked with a range of outcomes on main TSS usage

During yeast gametogenesis, many noncoding RNAs and mRNA isoforms are expressed. A class of transcripts called long undecodable transcript isoforms (LUTIs) initiate upstream of canonical promoters and are widely expressed [17–19]. A well-studied gene regulated by a LUTI is the kinetochore component *NDC80* [17, 18]. During early gametogenesis, transcription from the main *NDC80* TSS is repressed by transcription through the *NDC80* promoter, which initiated from the upstream alternative TSS (*NDC80*^*LUTI*^). Additionally, many other examples where transcription of intergenic noncoding RNAs or 5′ extended mRNA isoforms repress downstream promoters of protein coding genes have been reported [17, 18, 26, 28, 32, 55, 56]. While many LUTIs and noncoding RNAs have been functionally dissected and characterized, a more systematic analysis of how transcription from upstream alternative transcription isoform influences gene expression has been lacking. Close interrogation of our high-resolution time course allowed us to capture these regulatory events.

**Fig. 3:**
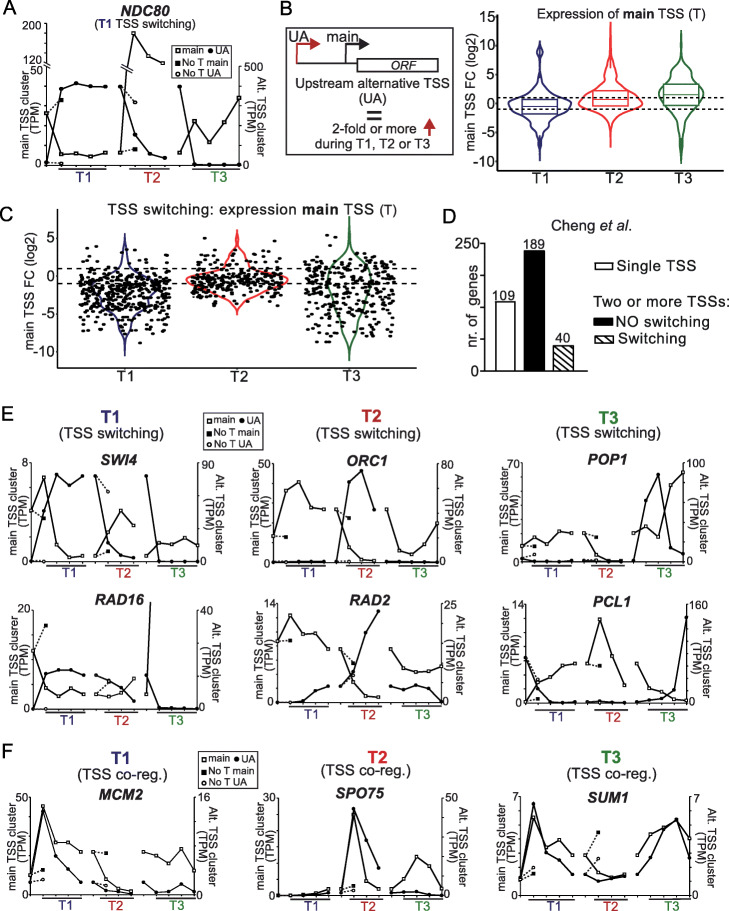
Increased upstream alternative TSS usage has varying effects on expression from the main TSS. **a** Main and upstream alternative TSS expression changes at the *NDC80* locus during T1, T2, and T3. Negative controls representing mock-treated samples for T1 and T2 (no induction of transition (No T) for main TSS and upstream alternative TSS (UA)) were included. The *y*-axis represents TPM values of the main and alternative TSSs. **b** Schematic of main and upstream alternative (UA) TSS pairs relative to their associated ORF (left). A subset of genes in which the alternative TSS was strictly upstream of the main TSS was used for the analysis. Increased expression from upstream alternative TSSs was linked to different outcomes on expression of the downstream main TSS (Right). Violin and overlaid boxplots showing increased expression from upstream alternative TSSs was linked to different outcomes on expression of the downstream main TSS. The dataset was drawn from upstream alternative TSSs paired with a downstream main TSS of the same gene. Observations were only included if the upstream alternative TSS was upregulated by twofold or more (FDR < 0.05), and the log_2_ fold change of the downstream main TSS was plotted on the *y*-axis. “T1” refers to transition 1 and represents the following comparisons: 3 h, 4 h, 5 h, or 6 h vs 2 h SPO. “T2” refers to transition 2 and represents the following comparisons: 7 h, 8 h, or 9 h vs 6 h SPO. “T3” refers to transition 3 and represents the following comparisons: 15 min, 60 min, or 120 min YPD vs 6 h SPO. The horizontal dashed lines mark log_2_ fold changes of 1 or − 1. The data represents TSS pairs from 546, 565, and 460 genes from T1, T2, and T3 respectively, and is an average of 3 biological repeats. **c** Violin plots representing log_2_ fold changes of main TSSs at the same time when a “TSS switching” event occurred. A switching event was counted when upstream alternative TSS expression increased by twofold or more (transition time point vs prior transition, FDR < 0.05). In addition, the TPM of an upstream alternative TSS had to be greater or equal to that of the downstream main TSS (TPM) at the same time point. The log_2_ fold change of the downstream main TSS was plotted on the *y*-axis. “T1” refers to transition 1 and represents the following comparisons: 3 h, 4 h, 5 h, or 6 h vs 2 h SPO. “T2” refers to transition 2 and represents the following comparisons: 7 h, 8 h, or 9 h vs 6 h SPO. “T3” refers to transition 3 and represents the following comparisons: 15 min, 60 min, or 120 min YPD vs 6 h SPO. The horizontal dashed lines mark log_2_ fold changes of 1 or − 1. The data represents TSS pairs from 109, 93, and 86 genes from T1, T2, and T3 respectively and is an average of 3 biological repeats. **d** The number of TSS switching events in a set of 380 genes were selected from Cheng et al. [19]. The number of genes with a single TSS is displayed. In addition, the number of genes with multiple TSSs with TSS switching events are shown. **e** Main and alternative TSS expression changes for example loci. Depicted are TSS switching events for *SWI4*, *ORC1*, *POP1*, *RAD16*, *RAD2*, and *PCL1*. **f** Similar as **e**, except that co-regulation events are displayed for *MCM2*, *SPO75*, and *SUM1*

Our TSS-seq data was consistent with our previous work on the *NDC80* locus, indicating that we can identify these regulatory events genome wide [17, 18] (Fig. 3a). During T1, the *NDC80* upstream alternative TSS was strongly upregulated and concomitantly the main TSS in the *NDC80* promoter was downregulated, while in T2 and T3, the TSS switching effects were reversed. Cells that were not induced for T1 and T2 but exposed to sporulation medium for the same time (Fig. 3a, “no T main” and “no T UA”, 3 h in SPO for T1 and 7 h in SPO for T2, respectively) did not display TSS usage changes at the *NDC80* locus, demonstrating that these effects are cell fate specific.

Having established that we could capture gene regulation events accompanying alternative TSS expression, we next examined how increased alternative TSS usage corresponded with expression changes from the matching downstream main TSS. We selected genes that showed upregulation (2-fold or more) of an upstream alternative TSS for at least one time point during the cell-fate transition. Surprisingly, expression from main TSSs changed with various outcomes in response to increased expression from an alternative TSS (Fig. 3b). For example in T1 at 3 h in SPO, 153 genes were downregulated, 87 genes were upregulated, and 184 genes did not change significantly. Genes in T2 and T3 showed a similar trend, but a slightly greater proportion of them were upregulated in expression (Fig. 3b). Genes with co-upregulated main-alternative TSS pairs in T1 and T3 were enriched for cell-fate transition specific biological processes (e.g., “double-strand break repair” during meiotic prophase (T1) and “ribosome biogenesis” during vegetative growth (T3)) (Additional file 8: Table S2). Downregulation of the main TSS in the presence of increased upstream alternative usage was not generally linked to specific biological processes. In contrast to increased alternative TSS usage, downregulation (2-fold or more) of some alternative TSSs was accompanied by downregulation of the main TSS, which suggests that some of these pairs of main and alternative TSSs could be co-regulated (Additional file 1: Fig. S4a). Additionally, at closely spaced tandem pairs of genes (< 200 bp apart), there was no clear effect of increased expression of upstream adjacent genes on expression of the main TSS of the downstream gene (Additional file 1: Fig. S4b). We conclude that upstream alternative TSS usage correlates with a range of effects on main TSS usage, from gene activation to gene repression. While our analysis does not establish a direct causative effect, our data suggest expression from the main TSS for many genes is influenced by transcription from upstream alternative TSSs, as reported in single-locus studies.

### TSS switching events are linked to various gene regulatory outcomes

Switching between main and alternative TSSs is reminiscent of the regulation we previously described at the *NDC80* locus (Fig. 3a, T1), where the alternative upstream transcript becomes the dominantly expressed isoform. To profile the effect on expression from the main TSS during such TSS shifts, we defined TSS switching by selecting TSS pairs where the alternative TSS is upregulated (2-fold or more), and its expression level must be at least equal or more than that of the main TSS (Fig. 3c). Across all transitions, we identified 109, 93, and 86 genes with TSS switching events in T1, T2, and T3 respectively. TSS switching events were linked to various degrees of downregulation of the main TSS. For example, the main TSSs of *NDC80* displayed a decrease of 5-fold in presence of expression from alternative upstream TSS, while the majority of main TSSs displayed a marginal decrease (2 folds or less).

We also assessed how previously reported LUTI-regulated genes (380 genes in total) behaved in our dataset (Fig. 3d) [19]. For a large fraction of genes (109 out of 380 genes) regulated by LUTI, we did not detect an alternative upstream TSS. It is possible that some alternative TSSs were not measured in our dataset because of technical limitations. For example, initiation of transcription from alternative TSSs could be spread over a large region in promoters, making it less sensitive for detection by TSS-seq. Surprisingly, the majority of previously defined LUTI-regulated genes (189 genes) that harbored an alternative TSS in our dataset displayed no TSS switching (Fig. 3d). This suggests that either most LUTI-regulated genes do not switch expression from protein coding TSS to the LUTI TSS. As a caveat, we cannot rule out the impact of noise in the TSS-seq data for the examples in which we observed little to no change in main TSS signals in the presence of increased upstream alternative TSS expression. Nevertheless, our analysis indicates that increased expression from upstream alternative TSSs is linked to various outcomes on the expression of the matching main TSSs and is not always associated with gene repression.

### TSS usage changes are dynamic and temporal

Gene regulation via expression from alternative TSSs is dynamic and cell-fate transition specific. Like *NDC80*, *SWI4* and *POP4* exhibited TSS switching in T1 (Fig. 3e, Additional file 1: Fig. S4c and S4d). At these loci, an upstream alternative TSS was upregulated, and the main TSS was downregulated concomitantly during T1. In T2 and T3, *SWI4* and *POP4* TSS switching was rapidly reversed. In comparison, the *RAD16* and *CLB2* genes showed a different switching pattern. Predominance of the alternative TSS after switching was maintained till the end of T2, indicating that expression from the alternative TSS could persist over multiple cell-fate transitions. We observed T2-specific switching for *ORC1* and *RAD2*, while *POP1* and *PCL1* showed strong switching events during T3 (Fig. 3e and Additional file 1: Fig. S4c). These examples illustrate that TSS switching not only occurs in a stage-specific manner but can also be spread across multiple stages (e.g., *RAD16* and *CLB2*) or controlled within a tight developmental window (e.g., *POP1* and *PCL1*).

Co-regulation of isoforms also occurs in a stage-specific manner (Fig. 3f, Additional file 1: Fig. S4c and Fig. S4d). Representative examples are the *MCM2* and *BDF2* genes where the main and alternative TSSs were both upregulated in T1 and then downregulated in T2. A similar pattern was observed for the *SPO75* and *SWD1* genes except that the alternative and matching main TSSs were co-upregulated in T2 (Fig. 3f, Additional file 1: Fig. S4c and S4d). At the *SUM1* locus, the expression of the main and alternative TSSs followed each other throughout all three fate transitions. Thus, the regulation of alternative-main TSS pairs is dynamic and can be coupled to shape gene expression at specific time points.

### Cell-fate transitions feature increased TSS usage within gene bodies

**Fig. 4:**
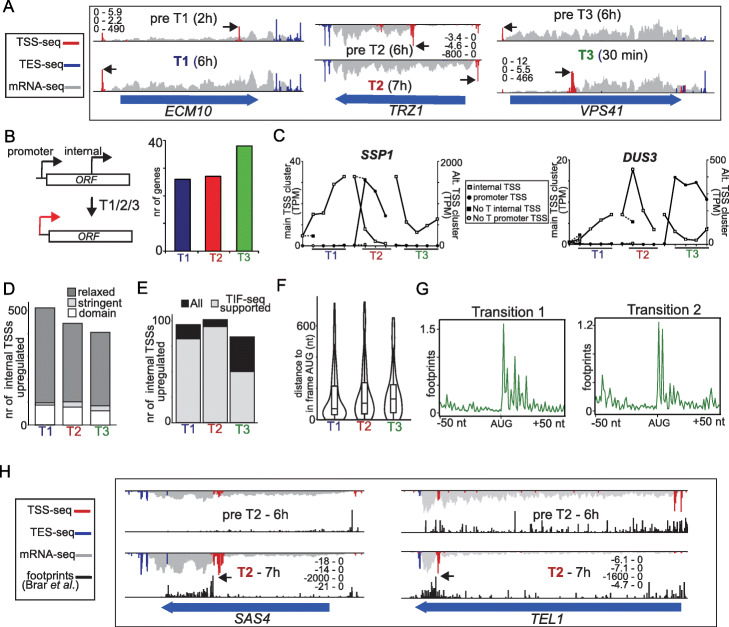
Widespread dynamic regulation of alternative TSSs within ORFs during cell-fate transitions. **a** Integrative Genomics Viewer (IGV) tracks showing examples of internal TSSs whose levels changed during cell-fate transitions (T1, T3, and T3). The scale for TSS-seq, TES-seq, and mRNA-seq values are displayed for *ECM10*, *TRZ1*, and *VPS41*. **b** Number of TSS switching events of genes that showed expression of main TSSs within the ORF sequences and which showed TSS switching during T1, T2, or T3 to canonical TSSs in the promoter sequence. Switching events were defined as in Fig. 3c. **c** Examples of switching events between TSSs expressed in ORFs (internal TSSs) and TSSs in promoters. Negative controls representing mock-treated samples for T1 and T2 (no induction of transition (T) for main TSS and upstream alternative TSS (UA)) were included. The *y*-axis represents TPM values of the internal and promoter TSS. **d** Numbers of TSSs expressed within ORFs (internal TSSs). Different cutoffs were used to define internal TSSs. Relaxed cutoff: internal TSSs increasing by 2-fold or more during transition. Stringent cutoff: internal TSSs belonging to putative transcripts encoding for an ORF that is at least 300 nucleotides in length and whose expression levels are at least one third that of the full-length mRNA at the same time point. Domain cutoff: internal transcripts with a predicted PFAM domain. The “stringent” and “domain” categories are subsets of the “relaxed” category. **e** Numbers of internal TSSs (stringent cutoff) identified by TSS seq, supported by transcripts with 5′ ends identified by TIF-seq. **f** The distribution of 5′ UTR length for the associated transcripts originating from internal TSSs approximated by computing the distance to the first in-frame AUG. *n* = 96/101/84 TSSs for T1/T2/T3 respectively. **g** Meta-profiles of ribosome footprints for internal TSSs (stringent cutoff). The ribosome footprint dataset was from Brar et al. [21]. **h** Examples of genes (*SAS4* and *TEL1*) that display expression from an internal TSS and whose internal transcripts are bound by ribosomes. The data for, and scales for TSS-seq, TES-seq, mRNA-seq, and the matching time-point for the ribosome footprint dataset are shown

TSS switching events were not limited to “conventional” promoter regions only but also occurred in regions downstream of the main TSS (Additional file 1: Fig. S2, labeled “internal”). We observed a subset of genes that displayed expression of a TSS within the coding sequence. Among these, about 30 to 40 genes showed transition-specific TSS switching, where the internal TSS was expressed prior to the transition but decreased during the transition while expression of the upstream TSS encoding for the full-length transcript concomitantly increased (e.g., *ECM10*, *TRZ1*, and *SPO22*,) (Fig. 4a, b and Additional file 1: Fig. S5a). We identified examples of genes where an internal TSS was upregulated during cell-fate transitions (e.g., *SSP1* and *DUS3*), and dynamic switching occurred between the full-length transcript and the internal TSS in a cell-fate-specific manner (Fig. 4c).

The production of truncated transcripts and protein isoforms via internal transcription during T2 was also reported previously [14]. To systemically dissect how internal TSSs are regulated across the different cell-fate transitions, we classified internal TSSs with relaxed (two-fold or more upregulated) and stringent cutoffs for each fate transition (Fig. 4d). The stringent cutoff was met when the expression levels were at least one third that of the full-length mRNA at the same time point, and the matching truncated transcript isoform contained an ORF that was more than 300 bp long (e.g., *VPS41*) (Fig. 4a). Nearly 500 internal TSSs were induced per transition, of which a substantial fraction remained after a stringent cutoff (Fig. 4d). The expression of internal TSSs was also supported by our TIF-seq dataset (Fig. 4e). Additionally, a subset of truncated transcript isoforms overlapped with coding sequences of specific protein domains, suggesting that encoded truncated proteins may have specialized cellular functions (Fig. 4d, labeled “domain”).

Several studies in yeast have shown that cryptic promoters exist within gene bodies, driving expression of short transcript isoforms and can encode for truncated proteins [14, 26, 57–63]. In our dataset, we found that many transcripts emanating from internal TSSs harbored an in-frame AUG not far from the internal TSS (Fig. 4f). Ribosomes were associated with truncated transcript isoforms initiating from internal TSSs when we compared a ribosome profiling dataset that covered the T1 and T2 cell-fate transitions with our dataset (Fig. 4g) [21]. For example, *SAS4* and *TEL1* showed clear ribosome footprint signals in the same region and at the same time when the truncated transcript isoform was expressed (Fig. 4h). Interestingly, the truncated transcript isoform of *TEL1* solely covered the FATC domain of the Tel1 protein, a domain that is important for mediating protein-protein interactions (Fig. 4h). Like *TEL1*, *TOR1*, the catalytic subunit of TORC1 and TORC2 in yeast, also showed expression of truncated transcript isoform solely encoding for the Tor1 FATC domain (data not shown).

Promoters controlling transcription from internal TSSs shared features with canonical promoters. We observed nucleosome-free regions (NFR) aligned with the internal TSS, and nucleosome periodicity (+ 1, + 2 nucleosomes and so on) downstream of the internal TSS (Additional file 1: Fig. S5b). Like the co-expression modules for T1 and T2, we found that the transcription factors Ume6 and Ndt80 were enriched upstream of internal TSSs (Additional file 1: Fig. S5c). Importantly, the expression of internal TSSs relied on the induction of Ime1 and Ndt80 expression, indicating that these internal transcripts are directly regulated by these transcription factors (Additional file 1: Fig. S3d). The promoter sequences of internal TSSs upregulated in T3 were enriched for the Sfp1 motif, suggesting that this transcription factor regulates truncated mRNA isoforms during return to the mitotic cell cycle (T3). Thus, similar to transcription upstream of canonical TSSs, the expression of transcripts with internal TSSs is also dynamically controlled, possibly by the same transcription factors that regulate the former. The short transcript isoforms, in turn, have the potential to be translated into truncated protein isoforms, diversifying the proteome during cell-state changes.

### Determinants of gene regulation via the use of alternative TSSs

Our analysis showed that increased upstream TSS usage is associated with a range of effects on expression of the downstream main TSS (Figs. 3b and 4b). Are there features in the dataset that can explain these outcomes? To examine this systematically, we aggregated the data obtained from three pairs of comparisons representing each cell-fate transition: T1 (6h vs 2 h SPO), T2 (8h vs 6 h SPO) and T3 (60 min YPD vs 6 h SPO). We focussed on two features in the dataset: alternative TSS levels and distance between alternative-main TSS pairs, as both features have been described to affect gene expression, in multiple studies [34, 42, 64].

**Fig. 5:**
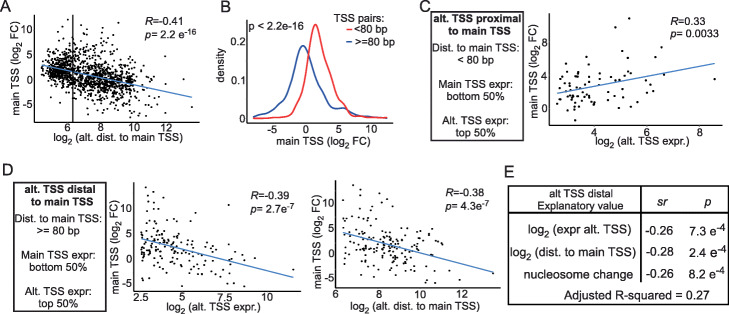
Features explaining main TSS usage changes upon increased alternative TSS usage levels. **a** Scatter plot of main TSS expression changes (transition versus prior transition state, log_2_, fold change) against distances between the upstream and downstream TSS (log_2_, nucleotides). The data presented is for T1 (6h vs 2 h in SPO), T2 (8h vs 6 h in SPO), and T3 (60 min YPD vs 6 h SPO) for a total of 1173 data points. A vertical line indicates the distance of 80 bp between main and upstream alternative TSSs. The Pearson correlation coefficient and its *p* value are displayed. **b** Density plots of main TSS expression changes (transition versus prior transition state, log2, fold change). The data was taken from three comparisons representing T1 (6h vs 2 h in SPO), T2 (8h vs 6 h in SPO), and T3 (60 min YPD vs 6 h SPO). The red density plot indicates main and upstream alternative TSSs pairs with < 80 bp distance between them (382 pairs), while the blue plot indicates the pairs with ≥ 80 bp distance between them (791 pairs). Main TSS changes from these two groups follow different distributions (Kolmogovov-Smirnov test, *p* < 0.05). **c** Scatter plot of main TSS expression changes (transition versus prior transition state, log_2_, fold change) against expression levels of alternative TSS (log_2_). Main and upstream alternative TSS pairs selected for alternative TSS which were proximal to the main TSS (< 80 bp), the alternative TSS value after transition was relatively high (≥ 50th percentile), and the main TSS value prior to transition was relatively low (≤ 50th percentile), representing 78 pairs. **d** Same as **a** and **b**, except that the data only includes genes whereby the alternative TSS is distal (≥ 80 bp) to the main TSS, the alternative TSS value after transition is relatively high (≥ 50th percentile) and the main TSS value prior to transition is relatively low (≤ 50th percentile), representing 164 pairs. **e** Multiple regression explaining fold change of main TSSs during cell-fate changes (*Y* = log_2_) by different variables. The genes chosen for this model fit the criteria in **d**, representing 164 pairs. The three different explanatory variables are given. The semi-partial correlation coefficients (*sr*) represent the strength of the linear relationship between *Y* and the specific explanatory variable that remains after controlling for the effects of the other explanatory variables (i.e., the unique effect). The *p* values reported in the table are for the unique effects of the explanatory variables

We found that main and alternative TSSs in close proximity were more likely to be co-regulated. For closely spaced alternative and main TSS pairs of less than 80 bp apart, increased alternative TSS usage correlated with increased main TSS usage, while expression of more widely spaced alternative and main TSS pairs correlated inversely instead (Fig. 5a, b). Moreover, genes which had shorter distances (< 80 bp) between the tandem TSSs displayed a positive correlation between alternative TSS expression levels and main TSS expression changes (Additional file 1: Fig. S6a). This correlation was strengthened at genes with relatively low main TSS expression prior to transition (≤ 50th percentile), and a relatively high alternative TSS expression during transition (≥ 50th percentile) (Fig. 5c and Additional file 1: Fig. S6a). The positive trend was weakened or even absent when we relaxed the criteria for the alternative TSS and main TSS expression levels (Additional file 1: Fig. S6a). This suggests that co-regulation between closely spaced TSSs occurs mostly when the expression from the alternative TSS is relatively high.

Second, we observed that expression from upstream alternative TSSs was linked to repression of the main TSS at genes when the distance between main and upstream alternative TSS was relatively large (≥ 80 bp) (Fig. 5d, Additional file 1: Fig. S6a and S6b). A stronger negative correlation between alternative TSS expression levels and main TSS expression changes was observed when we subsetted for genes with relatively low main TSS expression (≤ 50th percentile) and high alternative TSS expression (≥ 50th percentile) than without subsetting (Fig. 5d, left graph and Additional file 1: Fig. S6b). For this subgroup of genes, increasing distances between main TSS and alternative TSS were correlated with repression of the main TSS expression (Fig. 5d, right panel). The negative relationship weakened when we relaxed the alternative and main TSS expression levels and was absent when upstream alternative and main TSS were spaced 80 bp or less (Additional file 1: Fig. S6b and Fig. S6c). Our analysis suggests that the distance between main and alternative TSS, and alternative TSS expression levels are key determinants that influence how the main TSS responds when upstream TSS usage is increased.

The distance between TSSs was also reflected in the chromatin structure and transcription factor binding. Genes with a TSS of less than 80 bp upstream of the main TSS tended to have wider NFR and a defined peak for transcription factor binding (Additional file 1: Fig. S7a). On the other hand, genes with an alternative TSS of more than 80 bp upstream from the main TSS tended to have narrower NFRs around the main TSS, a second NFR nearby the upstream TSS, and displayed a broader peak for transcription factor binding. It is worth noting that upstream alternative TSS did not show a clear enrichment for the TATA binding sequence, suggesting that their transcription is regulated via TATA-less promoters.

### A linear model for gene regulation by upstream alternative TSSs

At genes with relatively large distance between alternative and main TSS, we observed that increased alternative TSS usage and further increase in distance between main and alternative TSS was linked to repression of the main TSS (Fig. 5d). Transcription initiating upstream of canonical promoters is known to alter chromatin state and represses promoters in numerous examples [18, 26, 28, 29, 65]. We found that changes in nucleosome occupancy in the region between the main and alternative TSS were consistent with decreased main TSS expression (Additional file 1: Fig. S7b). To dissect these different variables, we performed multiple regression analysis that accounts for relationships between different explanatory variables. This allowed us to delineate the semi-partial correlations (*sr*) between the response variable (main TSS levels) and a specific explanatory variable (e.g., nucleosome occupancy) (Fig. 5e)**.**

We identified a negative correlation between main TSS expression changes and upstream expression levels (first variable). The distance between the two promoters (second variable) also negatively correlated with our response variable likely because we already subsetted for genes’ relatively large (≥ 80 bp) distances (Fig. 5d). The combined model revealed that increased nucleosome occupancy was negatively correlated with changes in expression of the main TSS (Fig. 5e). Collectively, these three variables explained a significant part of the variation (adjusted R-squared = 0.27) in main TSS responses across the three cell-state transitions. We propose that a balance between expression levels of different TSSs of the same gene, the distance between tandem TSSs, and chromatin structure are key determinants for the regulation of gene expression via transcription of upstream alternative TSSs.

### Transcriptional repression via upstream alternative TSSs requires specific chromatin regulators

Our analysis and modeling did not establish causative relationships between upstream transcription, repression of transcription from the main TSS, and chromatin state. If the repression of the main TSS and changes in chromatin structure were the consequence of upstream transcription, certain chromatin factors are likely required for mediating gene repression. Disrupting chromatin factors may therefore affect the extent of repression driven by transcription from upstream alternative TSSs. Indeed, several regulators for chromatin have been described in facilitating repression via transcription of upstream noncoding RNAs or 5′ extended transcript isoforms [18, 26, 28, 29, 31, 66]. These include Set2-directed histone lysine 36 methylation, histone deacetylation directed by SET3C, and chromatin assembly by FACT.

**Fig. 6:**
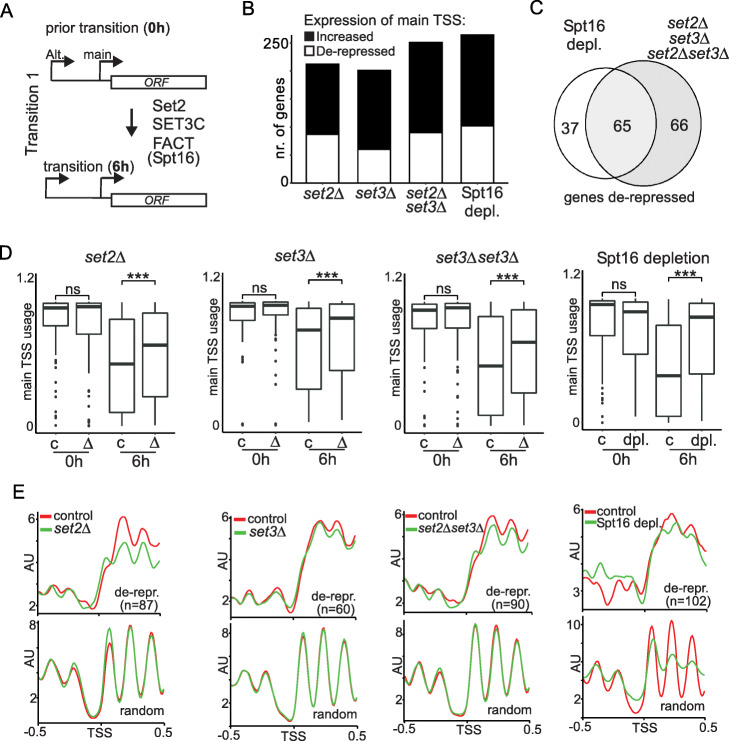
Chromatin factors mediate repression exercised by upstream alternative TSSs. **a** Schematic for determining how chromatin factors (Set2, Set3, and FACT) contribute to repression of the main TSS through transcription from alternative upstream TSSs. In short, cells harboring deletions (*set2Δ* (FW5767), *set3Δ* (FW5770), and *set2Δ set3Δ* (FW2912)) or depleted for Spt16 deletion (FVW6083) were compared to the control (FW2795) at the end of transition 1 (SPO 6 h). All these cells also had the *pCUP-IME1* and *GAL4.ER pGAL-NDT80* alleles. The Spt16 depletion allele (*SPT16-AID,* FVW6083) harbored *pCUP-TIR1*, while its matching control (FW6109) did not. Genes with upstream alternative TSSs were considered for this analysis. **b** Numbers of genes that displayed increased relative expression of the main TSS (black) or showed de-repression of the main TSS (white) (deletion or depletion mutants versus control). Genes within the de-repression category were selected for having increased relative expression of their main TSS in the different mutants and the main TSS was downregulated in the presence of expression of an upstream alternative TSS in control cells. The de-repression category is a subset of the “increased relative expression” category. Statistically significant changes in TSS usage were determined using the Cochran–Mantel–Haenszel (CMH) test on three independent repeats. **c** Venn diagram showing the number of genes de-repressed after Spt16 depletion and overlapped with genes de-repressed in the *set2Δ*, *set3Δ*, or *set2Δ set3Δ* deletion mutants. **d** Box and whisker plots of main TSS usage of genes under the de-repression category. The main TSS usage was calculated by dividing the main TSS value (TPM) over the sum of all TSSs values associated with the same gene for the same time point (0 h or 6 h in SPO). *** *p* < 0.0001. The number of data points corresponds to 87, 60, 90, and 102 genes for *set2*Δ, *set3*Δ, *set2*Δ*set3*Δ, and Spt16 depletion, respectively. **e** Meta-profiles of MNase-seq signals for *set2Δ*, *set3Δ*, and *set2set3Δ* cells or Spt16 depleted cells (green) and control cells (red) at the end of T1 (6h SPO). The signals were centered on the main TSS. The top panels show the profiles for de-repressed genes and the bottom panels for the same number of randomly selected genes (87, 60, 90, and 102 genes for *set2Δ*, *set3Δ*, *set2set3Δ*, and Spt16 depletion, respectively)

To test whether the chromatin state contributes to repression of transcription of the main TSS in the presence of upstream transcription, we generated deletion and depletion mutants and measured TSS usage and chromatin state (MNase-seq) during T1 (6h SPO) (Fig. 6a and Additional file 6). Importantly, cells harboring *set2*Δ and *set3*Δ single or double deletions entered meiosis and underwent premeiotic DNA replication, allowing for T1-specific transcriptome measurements (Additional file 1: Fig. S8a). Since FACT (Spt16) is essential for cellular growth, we depleted Spt16 using the auxin-induced degron (*SPT16-AID*) (Additional file 1: Fig. S8b). Importantly, these cells underwent premeiotic DNA replication even through Spt16 was depleted during entry into T1 (Additional file 1: Fig. S8c-e) [67].

To better capture locus-specific changes in gene expression in a backdrop of globally altered transcription in these chromatin mutants, we calculated the relative main TSS usage levels for each gene by dividing the main TSS signal over the sum of all TSSs associated with the same gene during T1 (6h SPO). Approximately 200 genes displayed increased main TSS expression in each of the deletion (*set2*Δ, *set3*Δ, and *set2*Δ*set3*Δ) and depletion (Spt16) mutants compared to WT (Fig. 6b). A subset of these genes showed significant de-repression of expression from the main TSS, indicating that chromatin regulators (Set2, Set3, and FACT) were required for mediating repression. We observed a good overlap between genes de-repressed after Spt16 depletion and genes affected by *set2*Δ and *set3*Δ single and double mutants (Fig. 6c). As expected, main TSS usage was significantly higher in T1 (6h SPO) among the genes identified as “de-repressed” in mutants compared to the control (Fig. 6d). Importantly, these differences were not observed prior to T1 (0h SPO) (Fig. 6d). Therefore, these de-repression events in these mutants occurred in the context of transition-specific gene regulatory programs. We posit that the failure to establish a repressive chromatin in the presence of upstream transcription results in leaky or aberrant expression from the main TSS.

We examined chromatin structure and observed a wider NFR near the main TSS during T1 in *set2*Δ and *set3*Δ single and double mutants, compared to the control at gene promoters that showed de-repression. This phenomenon was not observed at a matching number of randomly selected promoters. Depletion of Spt16 had a pronounced effect on chromatin structure at gene promoters that were de-repressed (Fig. 6e). We observed a wider NFR and the loss of regularly spaced nucleosome arrays flanking the main TSS, while the chromatin structure of randomly selected genes was disrupted to a lesser extent and regular nucleosome arrays were still visible. We further found that about 30% of depressed genes showed a significant occupancy change in chromatin structure of main TSS and 0.5 kb upstream. In conclusion, disrupting chromatin factors (Set2, Set3, and FACT) that mediate transcription coupled chromatin changes, affected the repression directed by transcription from upstream alternative TSSs, indicating that the effect of upstream transcription on repression of main TSS usage is direct.

### Messenger RNAs originating from upstream alternative TSSs have a variety of translation efficiencies

While there was a good overlap with genes expressing LUTIs and our TSS-seq dataset, we also found many genes that displayed expression from an upstream alternative TSS which were not identified as expressing LUTIs (Fig. 3). LUTIs are typically translationally inert due to the presence of small ORFs in their 5′ leader sequence [17, 19, 42]. Perhaps, a subset of transcripts produced from upstream TSSs have protein coding potential.

**Fig. 7:**
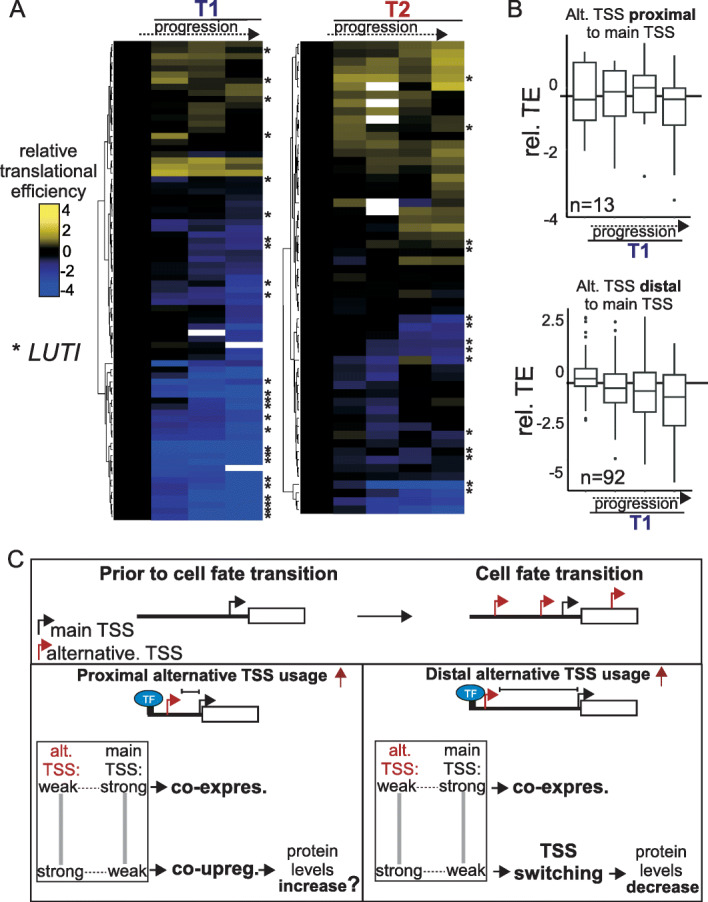
Genes expressing upstream alternative TSSs display a wide range of translational efficiencies. **a** Comparison of translation efficiencies (TE) across the cell-fate transitions T1 and T2 of genes that displayed a TSS switching event as described in Fig. 3c. TE values were obtained from Brar et al. [21]. We set the first time point of T1 (or T2) to 0 and other values being the TE differences between a later time point and the first time point. White colors indicate missing values. Asterisks after the gene labels indicate genes expressing LUTIs as defined by Cheng et al. [19]. **b** Box plots of TE values obtained from Brar et al. [21] for genes identified in T1 with TSS switching, separating into cases where the tandem TSSs were proximal to each other (< 80 bp, top panel) and cases where the tandem TSSs were distal to each other (≥ 80 bp, bottom panel). **c** Model for how transcription of the main TSSs and translational output are affected/regulated by alternative upstream TSSs. Top panel shows where alternative TSSs can be induced upon entering a cell-fate transition. Bottom panel shows how upstream alternative TSSs can influence expression from the main TSS during a cell-fate transition

To examine how upstream alternative transcripts are translationally controlled, we selected genes that underwent TSS switching, which ensures that the dominantly expressed transcript at these genes is initiated from the upstream alternative TSS (Fig. 3c). Subsequently, we examined translation efficiency using a published ribosome profiling dataset [21]. Consistent with previous work, we found a set of genes showing a decrease in translation efficiency as defined by ribosome footprinting (Fig. 7a) [19]. Many of these genes expressed LUTIs (Fig. 7a, genes marked with asterisks). This was particularly clear for the T1 transition, but less so for the T2 transition. A subset of genes showed no reduction in translation efficiency, suggesting that the upstream alternative transcript was translated (Fig. 7a). Interestingly, alternative TSSs that were more distal to the main TSS (≥ 80 bp) displayed decreased translational efficiency for T1, while proximal alternative TSSs showed no decrease (Fig. 7b). Perhaps, alternative TSSs proximal to the main TSS are less likely to harbor a small ORF in the 5′ leading sequence, while longer 5′ leading sequences reduce translation efficiency because of the presence of upstream small ORFs in the leading sequences which repress full-length protein production [42]. Interestingly, for T2, many genes showed no decrease in translational efficiency and there was little difference in translation efficiency between proximal and distal TSSs (Additional file 1: Fig. S8e). Our data suggest that the transcript isoforms emanating from upstream alternative TSSs possess different translation efficiencies.

## Discussion

In higher eukaryotes including human cells, mRNA isoforms are pervasively expressed often in a cell-type-specific manner [6, 65, 68]. Yet the regulatory significance of transcript isoform heterogeneity remains largely elusive. Our integrative genomic analysis of three distinct synchronized cell-fate transitions in yeast demonstrates that alternative TSS levels are pervasively upregulated during cell-fate transitions. Remarkably, expression from alternative TSSs located upstream in promoters was linked to expression of the main TSS with a range of outcomes, from co-regulation to repression.

### Alternative TSS usage is highly regulated during cell-state transitions

Our study indicates that TSS usage changes is highly pervasive and likely a key regulator of gene expression. First, a large fraction of genes showed upregulation of alternative TSSs when new cell-fate transitions were induced (Fig. 7c). Surprisingly, we found that transcription within coding regions was also induced, potentially coding for truncated protein isoforms. It is worth noting that we measured steady state levels of TSS usage, which could underestimate the number of TSSs. In particular, the TSSs from noncoding transcripts and transcripts with premature stop-codons or short upstream open reading frames are likely to be discarded rapidly via RNA degradation pathways [43, 69, 70]. Second, increased upstream alternative TSS usage correlated with a wide range of outcomes, ranging from repression to co-activation from the main TSS. Third, these outcomes can be explained by specific features in the data set. Alternative TSSs that were distal to main TSSs and were relatively highly expressed tended to repress expression from the main TSS. Conversely, alternative TSSs that were proximal to the main TSSs tended to be co-regulated. Fourth, differential TSS usage is highly dynamic, and reversible.

What drives changes in TSS usage? Our analysis suggests that specific transcription factors drive these usage changes. Alternative TSS usage depended on the expression of the transcription factors Ime1 and Ndt80 for T1 and T2 respectively. Specific motif sequences were enriched upstream of alternative TSSs. For example, the Ume6 motif and binding was enriched near alternative TSSs upregulated during T1, while the Tod6 and Sfp1 motifs were enriched proximal to those upregulated during T3. We propose that a combination of different transcription factors directs differential TSS usage.

### Multiple gene regulatory roles for increased alternative TSS usage

We provide evidence that upstream transcription influences transcription from the main TSS with a wide range of outcomes during all three cell-state transitions that we examined. Few genes displayed TSS switching behavior like at the *NDC80* locus that we had described previously [17, 18]. Thus, the *NDC80* gene is on the extreme of the spectrum of outcomes. The majority of genes displayed co-existence or co-upregulation of main and alternative TSSs. Although our TSS-seq data showed good reproducibility over three biological repeats (Additional file 2), we cannot rule out the role of experimental noise in why we observed co-existence of main and alternative TSSs for some gene loci. Further functional analysis and the use alternative techniques such as fluorescent in situ hybridization (FISH) or northern blot will be essential for studying the relationships between main and alternative TSSs more closely.

Previous work suggested that transcript isoform switching events are pervasive during yeast gametogenesis [19]. Many genes expressing long upstream transcript isoforms (LUTIs) displayed reduced protein levels compared to matching mRNA levels because ribosomes were stalled at the extended leader sequence [19]. The decrease in ribosome footprint signals was used to identify transcript switching events, and in this way many genes with transcript isoform switching were identified during yeast gametogenesis. While ribosome profiling can accurately measure translation efficiency of a given mRNA, it cannot discriminate between the isoforms produced. However, our TSS-seq analysis discriminated between the steady state levels of different transcript isoforms.

Even though many long isoforms are likely translationally inert, our analysis also suggests that upstream transcript isoforms may be translated, especially for genes where the alternative TSS is proximal to the main TSS (Fig. 7c). We propose that transcription from upstream transcript isoforms can have a wide range of outcomes on expression from the main TSS, and upstream transcript isoforms themselves are possibly translated with various translational efficiencies. We speculate that this multi-layered regulation allows for precise control of gene expression without the involvement of new regulatory proteins such as additional transcriptional repressors or transcriptional activators.

We also found that many TSSs were expressed and regulated in a cell-fate-specific manner within gene bodies. These internal transcripts have the potential to be translated into truncated protein isoforms because many of the internal TSSs were positioned directly upstream of an internal start codon and overlap with ribosome protected fragments and often covered conserved protein domains. Perhaps, short truncated proteins have specific functions during cell-state transitions. Several studies that have demonstrated that initiation of transcription within gene bodies generates truncated RNA isoforms [14, 58, 63, 71–73]. Yet, only a few studies have demonstrated a biological function of truncated protein isoforms [14, 74]. Our results warrant a more systematic approach to dissect the biological function of internal transcripts and short protein isoforms.

### Model for gene regulation by upstream alternative starts

Two features explain, at least in part, how alternative TSS usage is linked to transcription from the main TSS. First, the distance between two TSSs is a key consideration. If two TSS are close enough they are likely to be co-regulated, while more distal TSSs are regulated independently (Fig. 7c). One possibility is that transcriptional activators are shared between more proximal TSSs, but not at more distal TSSs. A threshold distance of 80 to 90 bp determines whether there is co-regulation, or more independent transcriptional control of two tandem TSSs. The 80 to 90 bp distance matches the minimum NRF region [75]. Perhaps, a distance greater than 80–90 bp allows chromatin assembly between two TSSs, generating two NFRs.

Secondly, expression levels of alternative and main TSSs play a determining role in the regulatory outcome caused by increased upstream alternative transcription. The higher the levels of the alternative upstream TSS, the more likely the repression of the main TSS (Fig. 7c). To achieve transcriptional repression of the main TSS during a cell-state transition, alternative TSSs are typically induced to higher levels than the main TSS prior to inducing the cell-fate transition. The repressive effect of alternative TSS depended on modifiers of chromatin (FACT, Set2, and SET3C) that act in the wake of transcription, suggesting a direct effect. We propose that the promoter activities for both alternative and main TSSs are critical for the regulatory outcome.

The balancing act between main and alternative promoter activities may also explain why transcription initiation can be induced within gene bodies during cell-state transitions. Normally, transcription initiation within gene bodies is repressed by transcription-coupled chromatin changes facilitated by transcription from the main TSS [76]. However, when transcriptional activators become more active during cell transitions, the promoter activity within ORF sequences increases. Subsequently, the repression exerted by the main TSS can be bypassed, and thus transcription initiation within gene bodies can occur, generating truncated RNA isoforms. With this view, alternative TSSs expressed upstream or downstream of the main TSS can shape gene expression in various ways.

Our data are consistent with previous observations. For example, different degrees of transcriptional repression of canonical transcript isoforms were also observed for a subset of genes expressing LUTIs [42]. Repression of sense mRNA transcription through the act of antisense transcription also has a level-dependent effect—the higher the antisense transcription, the stronger the repression of sense transcription [77]. A genome-wide interrogation of single cells of the mouse brain showed that many main and alternative TSS pairs tend to be co-regulated, in particular when closely spaced [78], suggesting conservation from yeast to mammalian cells.

### Concluding remarks

Our findings have broad implications on how alternative TSSs tune gene expression across all eukaryotes. Throughout development, alternative promoters and alternative TSSs are activated leading to increased transcript diversity [4]. Several single-locus studies have demonstrated that TSS switching events occur via mechanisms similar to that described in yeast [30, 79]. In addition, diseases such as cancers and neurodevelopmental disorders such as autism and epilepsy are associated with pervasive mis-regulation of alternative promoters [80, 81]. The regulatory principles of transcriptional control by differential TSS usage identified in this work could lead to a more systematic understanding of how transcript heterogeneity impacts development and disease.

## Methods

### Construction of yeast strains

All yeast strains used in this work were derived from the sporulation proficient SK1 strain background. The genotypes are listed in Additional file 7: Table S1. The genetic constructs bearing the *CUP1* promoter fusion with *IME1* (*pCUP-IME1*) and the *GAL* promoter fusion with *NDT80* (*pGAL-NDT80*) were described previously [40, 41]. Gene deletion strains were generated by a one-step promoter replacement protocol, and genetic crosses [82]. For inducing Spt16 depletion, we used the auxin-induced degron allele (AID) and a plasmid expressing *Oryza sativa osTIR1* ligase from a *CUP1* promoter (*pCUP1-TIR1*) (gift from Elçin Ünal) [67].

### Growth and medium conditions

For time course experiments, cells were grown in YPD (1.0% (wt/vol) yeast extract, 2.0% (wt/vol) peptone, 2.0% (wt/vol) glucose, supplemented with tryptophan (96 mg/l), uracil (24 mg/l), and adenine (12 mg/l), and grown to exponential phase at 30 °C and 300 rpm. Approximately 0.05 OD_600_ units of exponentially growing yeast were inoculated into new flasks containing reduced glucose YPD (1.0% (wt/vol) yeast extract, 2.0% (wt/vol) peptone, 1.0% (wt/vol) glucose), supplemented with uracil (24 mg/l) and adenine (12 mg/l). Cultures reached OD_600_ ≥ 10.0 after 16–18 h. Subsequently, cells were pelleted by centrifugation at room temperature (2000 *g*, 3 min), washed with sterile miliQ water, centrifuged again (2000 *g*, 3 min), and suspended in supplemented sporulation media (SPO) (1.0% (wt/vol) potassium acetate, supplemented with adenine/uracil (40 mg/l each), histidine/leucine/tryptophan (20 mg/l each), and 0.02% (wt/vol) raffinose) at OD_600_ of 2.5. For all experiments, cells were incubated in a shaker incubator at 30 °C and 300 rpm.

For the master time course, 50 μM CuSO_4_ was added after 2 h in SPO to induce *IME1* transcription from the *CUP1* promoter, which induces sporulation synchronously. After 6 h in SPO, 1 μM β-estradiol was added to induce *NDT80* expression, which in turn induces meiotic divisions and spore formation. To induce re-entry into the mitotic cell cycle, after 6 h in SPO, cells were pelleted by centrifugation at room temperature (2000*g*, 3 min) and resuspended in an equal volume of pre-warmed YPD.

To induce *SPT16-AID* depletion, cells expressing *SPT16-AID* and the *pCUP-TIR1* were grown to saturation in YPD as described above. Two hours after shifting to SPO, 50 μM CuSO_4_ was added to induce *IME1* and *TIR1* expression from the *CUP1* promoter while 500 μM indole-3-acetic acid (IAA) was added.

### FACS

The synchrony of pre-meiotic DNA replication was monitored by flow cytometry as previously described (BD LSR Fortessa, BD Biosciences) [41]. Cells were pelleted by centrifugation at room temperature (~ 2400*g*, 1 min) and fixed in 80% (vol/vol) ethanol for at least 60 min before further processing. Fixed cells were pelleted by centrifugation (~ 2400*g*, 1 min) and re-suspended in 50 mM Tris-HCl pH 7.5. Cells were sonicated for a few seconds before treatment with 0.2 mg/ml ribonuclease A in 50 mM Tris-HCl pH 7.5 at 37 °C overnight. After ribonuclease A digestion, cells were stained with 50 μg/ml propidium iodide in FACS buffer (200 mM Tris-HCl pH 7.5, 211 mM NaCl, and 78 mM MgCl_2_) for 1 h at room temperature before flow cytometry analysis. Propidium iodide-stained cells were excited with a 561 nm yellow-green laser and signals were detected using a 610/20 yellow filter. Pulse shape analysis (pulse height against pulse area) was used to exclude clumps and doublets. DNA content from single cells was estimated with a histogram of counts against pulse area. At least 50,000 cells were used for the analysis of each sample.

### DAPI staining

The rate of meiotic divisions was monitored by DAPI staining as previously described [41]. Cells were pelleted by centrifugation (~ 2400*g*, 1 min, room temperature) and fixed in 80% (vol/vol) ethanol for at least 60 min before further processing. Subsequently, samples were pelleted by centrifugation (~ 2400*g*, 1 min) and re-suspended in PBS with DAPI (1 μg/ml). Cells were sonicated for a few seconds and left in the dark at room temperature for at least 5 min. After DAPI staining, the proportion of cells containing one, two, three, or four DAPI masses were counted using a fluorescence microscope.

### Western blotting

Protein was extracted from cells with the V5 tagged *SPT16-AID* allele using methods as described previously [18]. Cells were pelleted by centrifugation at room temperature (~ 2400*g*, 1 min) and re-suspended in cold 5.0% w/v trichloroacetic acid (TCA) for at least 10 min. The pellets were then washed with acetone and mixed with lysis buffer (50 mM Tris pH 7.5, 1 mM EDTA, 2.75 mM dithiothreitol (DTT)), and cells were broken using a mini beadbeater (BioSpec). Lysates were mixed with SDS loading buffer (187.5 mM Tris pH 6.8, 6% v/v β-mercaptoethanol, 30% v/v Glycerol, 9% w/v SDS, 0.05% w/v Bromophenol Blue) and boiled for 5 min for denaturation. Proteins were separated by PAGE and transferred onto PVDF membranes using the Mini Trans-Blot Cell (Bio-Rad). The membranes were blocked for 60 min in blocking buffer (1.0% w/v BSA, 1.0% w/v milk) before incubation with mouse anti-V5 (R96025, Sigma-Aldrich) at a 1:2000 dilution overnight at 4 °C. Membranes were then washed in PBST (phosphate-buffered saline with 0.01% (v/v) tween-20) and incubated with anti-mouse HRP secondary antibodies at a 1:5000 dilution (GE Healthcare). After addition of ECL substrate (GE Healthcare), membranes were imaged using Imagequant 600 RGB (GE Healthcare).

For the Hxk1 loading controls, anti-hexokinase antibody (H2035, Stratech) was used at a 1:8000 dilution overnight at 4 °C. The IRDye 680RD donkey anti-rabbit secondary antibody (LI-COR) was used at a 1:15,000 dilution**.** Hxk1 levels were detected using an Odyssey Imager (LI-COR).

### RNA extraction

RNA was extracted for all time course experiments using previously described steps [18]. Up to 150 OD_600_ units of cells were collected, pelleted by centrifugation (~ 3000*g*, 3 min) and snap frozen in liquid nitrogen. For RNA extraction, frozen cells were re-suspended in TES buffer (10 mM Tris (pH 7.5), 10 mM EDTA, and 0.5% v/v SDS) and acid phenol:chloroform:isoamyl alcohol (125:24:1). At least 1 ml of TES and 1 ml of acid phenol:chloroform:isoamyl alcohol were used per 20 OD_600_ units of cells. Cell suspensions were heated on a thermomixer at 65 °C, with constant shaking at 1400 rpm for at least 45 min. Suspensions were centrifuged at 4 °C (> 10,000*g*, 5 min) and the aqueous phase was added to at least two volumes of ethanol with 0.3 M sodium acetate. RNA precipitation was done overnight at − 20 °C. RNA was pelleted by centrifugation at 4 °C (> 10,000*g*, 30 min), ethanol was removed by aspiration, and dried pellets were re-suspended in nuclease-free water (AM9932, Ambion).

### RNA sequencing

At least 2 μg of total RNA was treated with DNAse and purified on column (Macherey-Nagel). At least 400 ng of purified total RNA was used as input for the KAPA mRNA Hyper Prep kit (KK8580, Roche). Libraries were prepared according to manufacturer’s instructions. After bead-based clean up, libraries were sequenced on an Illumina HiSeq 2500 to an equivalent of 75 bases single-end reads, at a depth of approximately 20 million reads per library.

### Spike-in controls for TSS-seq and TES-seq

As quality check, we included spike-in controls for TSS-seq and TES-seq libraries as described previously [34, 83]. In short, a pool of in vitro transcripts (IVTs) were used spike-in controls for both TSS-seq, and TES-seq libraries were prepared as previously described [46]. Three different plasmids were used as templates for the in vitro transcription reaction. They were pGIBS-LYS (ATCC no. 87482), pGIBS-PHE (ATCC no. 87483), and pGIBS-THR (ATCC no. 87484). Each plasmid was linearized with NotI digestion for 1 h at 37 °C. Linearized templates were cleaned up with DNA columns (Macherey-Nagel). In total, 200 ng of each template was used for the in vitro transcription using 20 units of T3 RNA polymerase (P2083, Promega) in a reaction buffer (1X transcription optimized buffer (P1181, Promega), 10 mM DTT, 0.5 mM rNTP, and 0.5 μl RNasin Plus (N2611, Promega)), at 37 °C for 2 h. Subsequently, the DNA template was degraded using the TURBO DNA-free kit (AM1907, Ambion) at 37 °C for 30 min. IVTs were then extracted with acid phenol:chloroform:isoamyl alcohol (125:24:1) and precipitated in ethanol with 0.3 M sodium acetate. Quantification of IVTs was done using the Qubit RNA high sensitivity assay kit (Q32852, Thermo Fisher Scientific), and IVT sizes were checked using a Bioanalyzer (Agilent Technologies). The expected lengths of the pGIBS-LYS, pGIBS-PHE, and pGIBS-THR IVTs were 1106, 1407, and 2070 nucleotides respectively. Purified IVTs were pooled at an approximate molecular ratio of 25 Lys:5 Phe:1 Thr. One microgram of pooled IVTs was denatured at 65 °C for 5 min, cooled on ice, and then subjected to a capping reaction (10 U Vaccinia capping enzyme (M2080, NEB), 1X capping buffer (M2080, NEB), 0.5 mM GTP, 0.1 mM SAM (B9003S, NEB), and 0.5 μl RNasin Plus). Pooled IVTs were then extracted with acid phenol:chloroform:isoamyl alcohol (125:24:1) and precipitated in ethanol with 0.3 M sodium acetate.

### TSS-seq

TSS-seq protocol was adapted and modified [20, 43, 44] and described previously [34, 83]. In short, 50 ng of pooled IVTs were spiked into 1 mg of total RNA from each sample. At least 5 μg of mRNAs was purified from total RNA using the Poly(A)Purist MAG kit (AM1922, Ambion). mRNAs were fragmented, and fragments were dephosphorylated and then subjected to a decapping reaction using Cap-Clip acid pyrophosphatase (C-CC15011H, Tebu-bio). We also included a non-decapping control for some of the samples. Subsequently, a custom 5′ adapter (CACTCTrGrArGrCrArArUrArCrC) was ligated to the de-capped RNA, reverse transcribed, and was subjected to second-strand synthesis using second-strand biotinylated primer (GCAC/iBiodT/GCACTCTGAGCAATACC). Double-stranded product (dsDNA) was purified and at least 1 ng of dsDNA was then used as input for the KAPA Hyper Prep Kit (KK8504, Roche) and ligated to KAPA single indexed adapters Set A (KK8701, Roche) or Set B (KK8702, Roche). Library amplification was done on the biotinylated dsDNA fraction bound to the beads. Depending on the input amounts, 15–17 PCR cycles were used to generate libraries. Amplified libraries were quantified and purified. Purified libraries were on an Illumina HiSeq 2500 (75 bases single-end reads and approximately 20 million reads per library).

### TES-seq

The TES-seq library preparation were adapted from previously described protocols [45, 84], and were also described previously [34]. In short, from the same pool of fragmented mRNAs for each sample, at least 1 μg was used for TES-seq. RNA fragments were mixed with 2.5 μM GsuI20TVN primer (/5BiotinTEG/ GAGCTAGTTCTGGAGTTTTTTTTTTTTTTTTTTTTVN), 0.5 mM 5-Methylcytosine-dNTPs (D1030, Zymo Research), and 0.5 μl RNasin Plus. SuperScript IV was used for the reverse transcription reaction, and samples were purified, subjected to second-strand synthesis, and captured with MyOne Streptavidin C1 Dynabeads. Samples were eluted from beads following GsuI digestion (ER0461, Thermo Fisher Scientific). At least 1 ng of dsDNA was then used as input for the KAPA Hyper Prep Kit (KK8504, Roche) and ligated to KAPA single indexed adapters Set A (KK8701, Roche) or Set B (KK8702, Roche). Purified libraries were on an Illumina HiSeq 2500 (75 bases single-end reads and approximately 20 million reads per library).

### Ume6 ChIP-seq

Diploid cells suspended in SPO for 4 h were collected for chromatin immunoprecipitation (ChIP) experiments for the V5 tagged Ume6 transcription factor. Cells expressing untagged controls were included in the analysis. Cells were fixed in 1.0% w/v of formaldehyde for 15–20 min at room temperature and quenched with 100 mM glycine. After breaking cells using a mini beadbeater (BioSpec), crosslinked chromatin was sheared by sonication using Bioruptor (Diagenode, 6 cycles of 30 s on/off). Extracts were incubated with anti V5 agarose beads (Sigma) for 2 h, and beads were washed. Subsequently, reverse cross-linking was done in Tris-EDTA buffer (100 mM Tris pH 8.0, 10 mM EDTA, 1.0% v/v SDS) at 65 °C overnight. After 2 h of proteinase K treatment, DNA was purified on column (Macherey-Nagel). The concentration of purified DNA was quantified using the Qubit dsDNA HS assay kit (Q32851, Invitrogen). DNA was then used as input for the KAPA Hyper Prep Kit (KK8504, Roche) and ligated to KAPA single indexed adapters Set A (KK8701, Roche). Libraries were constructed according to the manufacturer’s instructions. Purified libraries were further quantified and inspected on a Tapestation (Agilent Technologies) and sequenced on an Illumina HiSeq 2500 to an equivalent of 50 bases single-end reads, at a depth of approximately 16 million reads per library.

### MNase-seq

We extracted mononucleosomes using a micrococcal nuclease (MNase) digestion protocol that was described previously [47, 85]. Approximately 90 OD_600_ units of cells were crosslinked at 30 °C for 20 min with formaldehyde (1% v/v) and the reaction was quenched with glycine (125 mM). Subsequently, cells were resuspended in 20 ml of buffer Z (1 M sorbitol, 50 mM Tris-HCl pH 7.4) plus β-mercaptoethanol (10 mM) and treated with 250 μg of T100 Zymolase (MP Biomedicals) at 30 °C for 60 min. Next, spheroplasted cells were pelleted (4000*g*, 10 min) and resuspended in 1 ml NP buffer (0.5 mM spermidine, 1 mM β-mercaptoethanol (β-ME), 0.075% (w/v) Tergitol solution-type NP-40 detergent (NP-40), 50 mM NaCl, 10 mM Tris-HCl pH 7.4, 5 mM MgCl_2_, 1 mM CaCl_2_). Separate aliquots of this lysate were treated with 400, 167, and 80 gel units of MNase (M0247S, NEB) for 30 min at 37 °C, and the reaction was quenched with EDTA (10 mM). In total, 100 μl of MNase treated and untreated extracts were reverse crosslinked at 65 °C overnight in 1 ml of SDS-TE (1.0% w/v SDS, 10 mM Tris pH 8.0, 1 mM EDTA, 4.5% v/v proteinase K). Extracts were then treated with 20 μl RNase A (10 mg/ml), DNA was extracted with phenol-chloroform-isoamyl alcohol (25:24:1) and precipitated at − 20 °C overnight in ethanol with 0.3 M sodium acetate. To check the extent of MNase digestion, purified DNA fragments were electrophoresed on a 2% agarose gel. The extracts which showed a mostly mono-nucleosome pattern were gel purified (Macherey-Nagel) and used as inputs for the KAPA Hyper Prep Kit (KK8504, Roche). Ligations were done using KAPA single indexed adapters Set A (KK8701, Roche) or Set B (KK8702, Roche). Libraries were constructed according to the manufacturer’s instructions. Purified libraries were sequenced on an Illumina HiSeq 2500 (100 bases paired-end reads and approximately 8 million reads per library).

### Transcript isoforms sequencing (TIF-seq) and bioinformatic analysis

The transcription isoform sequencing procedure was performed according the TIF-Seq2 protocol as described previously, except that we used 7.5 μg of total RNA for the starting reaction [86]. Libraries were sequenced on an Illumina NextSeq 500 (76-nt reads and approximately 14~65 million reads per library).

TIF-Seq2 analysis was performed as described previously with read mapping to the SK1 genome assembly [86]. The source code of the pipeline is available online (https://github.com/jingwen/TIFseq2). We then linked aligned paired-end reads and kept the uniquely mapped pairs that are in the same chromosome. We converted alignment BAM files to BEDPE format using “bedtools bamtobed”.

### Adapter trimming and read alignment

For the RNA-seq data, adapter trimming was performed with cutadapt (version 1.9.1) [87] with parameters “-a AGATCGGAAGAGCACACGTCTGAACTCCAGTCAC --minimum-length=20”. STAR (version 2.5.2) [88] with parameters “--alignIntronMin 3 --alignIntronMax 5000” was used to perform the read mapping to the *Saccharomyces cerevisiae* SK1 genome assembly from Keeney lab. Alignments with mapping quality of < 10 or soft/hard-clipping were filtered. The tool “bedtools genomecov” [89] was used to generate the RNA-seq coverage tracks across the genome.

For the TSS-seq and TES-seq, adapter trimming of raw reads was performed with cutadapt (version 1.9.1) with parameters “-a AGATCGGAAGAGCACACGTCTGAACTCCAGTCAC --minimum-length=20”. In addition, for the TSS-seq data, the custom 5′ adapter sequence specific to the protocol was removed by re-running cutadapt with the parameters “-g CACTCTGAGCAATACC -O 16 --minimum-length=20”, and only the reads containing the adapter sequence were used for further analysis. STAR (version 2.5.2) with parameters “--alignIntronMin 2 --alignIntronMax 1” (i.e., not allowing introns) was used to align TSS-seq and TES-seq reads to the SK1 genome assembly (plus three spike-in sequences). The alignments with mapping quality of ≥ 10 were kept for further analysis. For TSS-seq alignments, the 5′-most nucleotides of reads were extracted and the genome-wide coverage tracks were generated. For TES-seq alignments, we only kept the reads with soft-clipping at the 3′ end (size of soft-clipping part ≤ 10 bp) and required at least two consecutive non-templated As in the soft-clipping part. Insertions/deletions were also not allowed for the TES-seq alignments. The 3′-most nucleotides of aligned TES-seq reads were extracted, and genome-wide coverage tracks were generated. Since some 3′ end signals may be artifacts due to the poly(A) tracts in the gene body, we excluded the 3′ end sites which are overlapping or close to (≤ 5 nt distance) downstream poly(A) tracts (defined by a motif of (A)_20_ with ≤ 8 mismatches) from downstream cluster calling.

For the MNase-seq data, adapter trimming was performed with cutadapt (version 1.9.1) with parameters “-a AGATCGGAAGAGCACACGTCTGAACTCCAGTCA -A AGATCGGAAGAGCGTCGTGTAGGGAAAGAGTGT”. “bwa mem” (version 0.7.15) was used to perform the read mapping to the SK1 genome assembly. The resulting alignments were filtered by SAMtools (version 1.3.1) [90] with parameters “-q 10 -f 2 -F 2828.”

### Pairwise correlations between samples based on alignments

Using the genome-wide coverage tracks generated above, we calculated pairwise correlations between samples (between replicates or non-replicates) for TSS-seq, TES-seq, and RNA-seq data respectively. First, for each sample, we extracted the average coverage values for 100 bp non-overlapping widows across the genome using the command “multiBigwigSummary” in deepTools [91], for TSS-seq, TES-seq, and RNA-seq data respectively. The resulting coverage matrixes (TSS-seq, TES-seq, or RNA-seq) of 100-bp windows were used to calculated pairwise Pearson’s correlations between samples in R (version 3.4.1).

### TSS/TES cluster calling with CAGEr

The 5′-most (for TSS-seq data) and 3′-most (for TES-seq data) nucleotides were clustered into TSS or TES “clusters” using CAGEr [92]. The key parameters were (1) clusterCTSS: method = “distclu”, maxDist = 5, keepSingletonsAbove = 3, and (2) aggregateTagClusters: tpmThreshold = 1, qLow = 0.05, qUp = 0.95, maxDist = 20.

### Genome-wide differential expression analysis

Differential expression analysis was performed with the DESeq2 package (version 1.18.1) [93] in R (version 3.4.1). The raw read counts of called TSS/TES clusters, which are required input for running DESeq2, were extracted from aforementioned coverage tracks by bigWigAverageOverBed [94]. The clusters with log2(fold change) > =1 and padj < 0.05 in the DESeq2 results were considered as significantly differentially expressed clusters.

Each time point corresponding to early gametogenesis (SPO 3–6 h) was compared to pre-meiotic cells immediately prior to *IME1* induction (SPO 2 h). Each time point corresponding to mid-late gametogenesis (SPO 7–9 h) was compared to cells in meiotic prophase, prior to *NDT80* induction (SPO 6 h). The SPO 3M starvation controls were compared to pre-meiotic cells at SPO 2 h. The SPO 7M starvation controls were compared to meiotic prophase cells at SPO 6 h.

### TSS/TES assignment to gene features

The assignment of the TSS/TES clusters to their nearby genes was done by “bedtools closest” [89]. Assignment of TSSs to the nearest TES was also done by “bedtools closest.” Each TSS/TES was only assigned to a single closest, non-overlapping gene feature. To remove spurious TSSs, we sequenced a non-decapping control sample for the 5′ end data. We noticed that some TSS clusters which are located within the gene body also show enriched read signals in the non-decapping control, suggesting these clusters might not be genuine TSSs. To remove this type of potential artifacts, for each called TSS cluster, we calculated the Spearman correlation between the non-decapping sample and the 0 h sample using the base-wise read counts in the region of TSS cluster ± 5 bp. We excluded the suspicious 273 TSS clusters that show significant correlation between non-decapping sample and the 0 h sample (Spearman’s *r* > 0.5 and *p* < 0.05) from downstream analysis.

The remaining TSS clusters were first assigned to downstream gene features in the same orientation if they were within 100 bp from the start of the feature. Some TSS clusters are further than 100 bp but still within 1000 bp of a downstream gene feature in the same orientation. In such cases, a 30-bp window was slid from the TSS cluster to the start of the gene. These clusters were only assigned to the gene if median read counts in all the windows were greater than zero.

TES clusters were assigned to upstream gene features in the same orientation if they were within 100 bp from the end of the feature. Some TES clusters are further than 100 bp but still within 1000 bp of an upstream gene feature in the same orientation. In such cases, additional assignment criteria were adopted, modified from a previously described approach [11]. A 30-bp window was slid from the TES cluster to the end of the gene. These clusters were only assigned to the gene if median read counts in all the windows were greater than zero. In addition, the median read count in each window had to be greater than or equal to 5% of the median read count over the gene feature. Lastly, the maximum read count in each of the intervals had to be less than or equal to five times the maximum read count over the gene feature.

The output from “bedtools closest” was also used to determine the 5′ and 3′ UTRs length. The 5′ UTR length is defined as the distance given in number of nucleotides from the apex of a TSS cluster to the AUG of an annotated ORF. The 3′ UTR length is defined as the distance given in the number of nucleotides from the apex of a TES cluster to the stop codon of an annotated ORF. TSS/TES clusters were also assigned to genes if they were located within gene bodies in the same orientation.

### Main or alternative TSS/TES classification

The master time course was divided into three cell-fate transitions: T1, T2, and T3. T1 denotes pre-meiotic to meiotic prophase (SPO 2 h–6 h). T2 denotes prophase to meiotic divisions (SPO 6 h–9 h). T3 denotes return to mitotic cell cycle (SPO 6 h and 15–120 min after shifting cells to YPD). The main TSS/TES for each gene was defined as the CAGEr—predicted cluster with the highest expression (tags per million, TPM) and had ≥ 1 TPM in pre-meiotic cells for T1, and in prophase cells for both T2 and T3. Therefore, up to two main TSSs/TESs were defined for each gene, one for the pre-meiotic stage and another for the meiotic prophase stage. Alternative TSSs/TESs for each gene and for each transition were defined as any annotated non-main TSS/TES which had ≥ 1 TPM during at least one time point.

### Internal TSS classification and internal ORF prediction

For a TSS cluster to be classified as “internal” or assigned to a gene body, its apex must be within the genomic interval of an annotated ORF. To predict internal ORFs with higher confidence, all alternative internal TSSs which were upregulated by 2 fold or more during each transition were selected. In addition, these alternative internal TSSs were further filtered to retain only those whose expression levels were at least 33% that of main TSS, and by proxy, the main mRNA isoform. Next, putative internal coding features were defined from these candidate internal TSSs to the stop codon of the gene feature which they were embedded in. The coordinates of these internal coding features were then extracted into a BED format. FASTA nucleotide sequences of each feature were obtained using “bedtools getfasta,” using the SK1 genome assembly and the aforementioned BED file as inputs. These FASTA sequences were analyzed using the EMBOSS “getorf” tool, using the standard genetic code and a minimum ORF nucleotide size of 300 in the forward sequence only (http://www.bioinformatics.nl/cgi-bin/emboss/getorf) [95]. The output FASTA amino acid sequences were then passed to the HMMER “hmmscan” tool to detect conserved protein domains. The HMM database (hmmdb) used in this analysis was created using the hmmpress command on the Pfam 32.0 database [96].

### WGCNA co-expression analysis

For co-expression analysis, we used the normalized expression levels after variance stabilizing transformations from the DESeq2 analysis. We applied the “removeBatchEffect” function from the R package “limma” to account for the batch effects in the data. Next the “blockwiseModules” function in WGCNA (version 1.66) [48] was used to identify the co-expressed modules, with parameters “power = 18, networkType = ‘signed,’ TOMType = ‘signed,’ minModuleSize = 30, reassignThreshold = 0, mergeCutHeight = 0.25.” For each TSS co-expression, we used the tool “findMotifsGenome.pl” in the HOMER package (version 4.9.1) [97] to infer enriched motifs in the core promoter regions ([-150nt, 50nt] of TSSs), with parameters “-size -150,50 -mset yeast”.

### Gene ontology enrichment analysis

Lists of genes with increased upstream transcription at each transition were uploaded to the Gene Ontology (GO) knowledgebase (http://geneontology.org/). GO enrichment analysis was done using the PANTHER overrepresentation test (Released 20200407) [98]. Significantly enriched GO processes were determined using Fisher’s exact test (FDR < 0.05), using a reference list of all *Saccharomyces cerevisiae* genes in the PANTHER database.

### MNase-seq data analysis

Based on the MNase-seq read alignments, the DANPOS2 “dpos” command (version 2.2.2) [99] was used for generating the genome-wide occupancy tracks and identifying regions with significant changes, with parameters “-q 1000 -a 1 -m 1 --clonalcut 1e-10 --testcut 1e-10 --extend 74”. We extracted the regions showing significant changes in dyad position, fuzziness, or occupancy in different comparisons using the FDR cutoff of 0.1. For position shift, we required at least 5 nt distance between the two dyads in comparison. For the depressed genes, we calculated how many genes show significant occupancy differences between mutant and control samples (based on DANPOS2 results) in regions from the main TSS to 0.5 kb upstream. The numbers of depressed genes with significant occupancy changes are 32 (out of 87), 26 (out of 60), 26 (out of 90), and 27 (out of 102), for *set2*Δ, *set3*Δ, *set2*Δ*set3*Δ, and depletion (*SPT16-AID*) respectively.

### ChIP-seq data analysis

Ume6 ChIP-seq data were generated in the study, while Ndt80 ChIP-seq data were from a previous study (GEO accession GSE90661) [100]. For the Ume6 ChIP-seq data, adapter trimming was performed with cutadapt (version 1.9.1) [87] with parameters “--minimum-length=25 --quality-cutoff=20 -a AGATCGGAAGAGC,” while for Ndt80 ChIP-seq, the cutadapt parameters are “-a AGATCGGAAGAGC --minimum-length = 20.” BWA (version 0.5.9-r16) [90] with default parameters was used to perform genome-wide mapping of the adapter-trimmed reads to the SK1 genome. Duplicate marking was performed using the picard tool MarkDuplicates (version 2.1.1) (http://broadinstitute.github.io/picard). Further filtering was performed to exclude reads that were duplicates and ambiguously mapped.

The peak calling for Ume6 ChIP-seq data was done by the command “macs2 callpeak” in MACS2 [101] with parameters “-g 12000000 -m 3 100 -B -q 0.05.” For the Ndt80 data, the parameters of “macs2 callpeak” are “-g 12000000 --nomodel -B -q 0.1.” Based on the output of “macs2 callpeak,” the signal tracks of Ume6/Ndt80 ChIP-seq were generated by the “macs2 bdgcmp” command with parameters “-m ppois” [89, 94, 97].

### Multiple regression analysis

To generate the dataset of main and alternative TSSs for the multiple regression analysis, several selection criteria were applied. The TSSs must be associated with a gene, with ≥ 1 TPM expression prior or during the transition. Only alternative TSSs which were upstream of the main TSSs, and which were external to the ORF were included. In addition, expression from the alternative TSS must be upregulated by two-fold or more during the transition predicted by DESeq2. T1 is represented by the 6 h vs 2 h comparison, T2 by the 9 h vs 6 h comparison and T3 by the 60 min vs 6 h comparison. In cases where genes were associated with multiple alternative TSSs, only one alternative TSS was randomly chosen for the modeling dataset. All regression models were fitted using the lm() function in R. The semi-partial correlation coefficients were calculated using the R package “ppcor” (version 1.1).

### Analysis of differential TSS usage in mutants

Raw read counts from TSS-seq were used to detect TSSs which are differentially used in mutant cells relative to control cells, during meiotic prophase. To facilitate comparisons between mutant and control cells, TSS clusters defined by CAGEr for the mutant samples were mapped to TSS clusters from control samples using “bedtools intersect.” TSS clusters unique to mutant samples were also retained and assigned to gene features using “bedtools closest.” TSS read counts from three independent biological repeats were organized into contingency tables and grouped by gene. The main pre-meiotic TSS read counts were compared with the sum of read counts from all cognate alternative TSSs for control and mutant cells. The Cochran-Mantel-Haenszel test (CMH) was applied to these data using the “mantelhaen.test” function from the “stats” package in R. Briefly, this tests for whether the proportion of main TSS reads for any gene during meiotic prophase in control cells is consistently different to that of mutant cells, across three biological repeats. Genes with increased main TSS usage in mutants relative to controls were shortlisted if the CMH test produced an adjusted *p* value < 0.05 and if the proportion of main TSS reads was larger in the mutants.

### Statistical analysis and data visualization

All statistical analyses were performed using R version 3.4.1 and above. Data visualization was carried out using the R packages ggplot version 3.3.1 and ggpubr version 0.4.0.

## Supplementary Information

**Additional file 1: Figs. S1-S8** and legends.

**Additional file 2.** Pairwise Pearson’s correlations between samples in the master time course, based on average read signals for 100 bp non-overlapping windows across the genome. Three separated sheets for TSS-seq, TES-seq and mRNA-seq data respectively.

**Additional file 3.** TSS-seq versus RNA-seq plots for all time points.

**Additional file 4.** TES-seq versus RNA-seq plots for all time points.

**Additional file 5.** TSS-seq versus TES-seq plots for all time points.

**Additional file 6.** Pairwise Pearson’s correlations between TSS-seq samples for deletion or depletion mutants (Set2 Set3, and Spt16), and corresponding controls, based on average read signals for 100 bp non-overlapping windows across the genome.

**Additional file 7: Table S1.** Yeast strain genotypes.

**Additional file 8: Table S2.** GO analyses.

**Additional file 9.** Review history.

## Data Availability

The sequencing data generated in the work have been deposited in NCBI’s Gene Expression Omnibus and are accessible through GEO Series accession number GSE137711 [102].
