# Author Correction: High-resolution analysis of cell-state transitions in yeast suggests widespread transcriptional tuning by alternative starts

**DOI:** 10.1186/s13059-021-02274-6

**Published:** 2021-01-25

**Authors:** Minghao Chia, Cai Li, Sueli Marques, Vicente Pelechano, Nicholas M. Luscombe, Folkert J. van Werven

**Affiliations:** 1 The Francis Crick Institute, London, UK; 2 Genome Institute of Singapore, 60 Biopolis Street, Genome, #02-01, Singapore, 138672 Singapore; 3 School of Life Sciences, Sun Yat-sen University, Guangzhou, China; 4 SciLifeLab, Department of Microbiology, Tumor and Cell Biology, Karolinska Institutet, Solna, Sweden; 5 Okinawa Institute of Science & Technology Graduate University, Okinawa, 904-0495 Japan; 6 UCL Genetics Institute, University College London, London, WC1E 6BT UK

**Correction to: Genome Biol 22, 34 (2021)**

**https://doi.org/10.1186/s13059-020-02245-3**

Following publication of the original paper [1], the authors reported an error in affiliation 2. The correct affiliation is as follows:

Genome Institute of Singapore, 60 Biopolis Street, Genome, #02-01, Singapore, 138672 Singapore

The original article [1] has been corrected.
