# Supplementary material for: High-resolution analysis of cell-state transitions in yeast suggests widespread transcriptional tuning by alternative starts
Source: Genome Biol. 2021 Jan 14;22:34. doi: 10.1186/s13059-020-02245-3 (PMC7807719; doi:10.1186/s13059-020-02245-3)
Supplement: Supplementary file 1 — Additional file 1: Figs. S1-S8 and legends. [file 13059_2020_2245_MOESM1_ESM.pdf]

Fig. S1 (Chia et al)

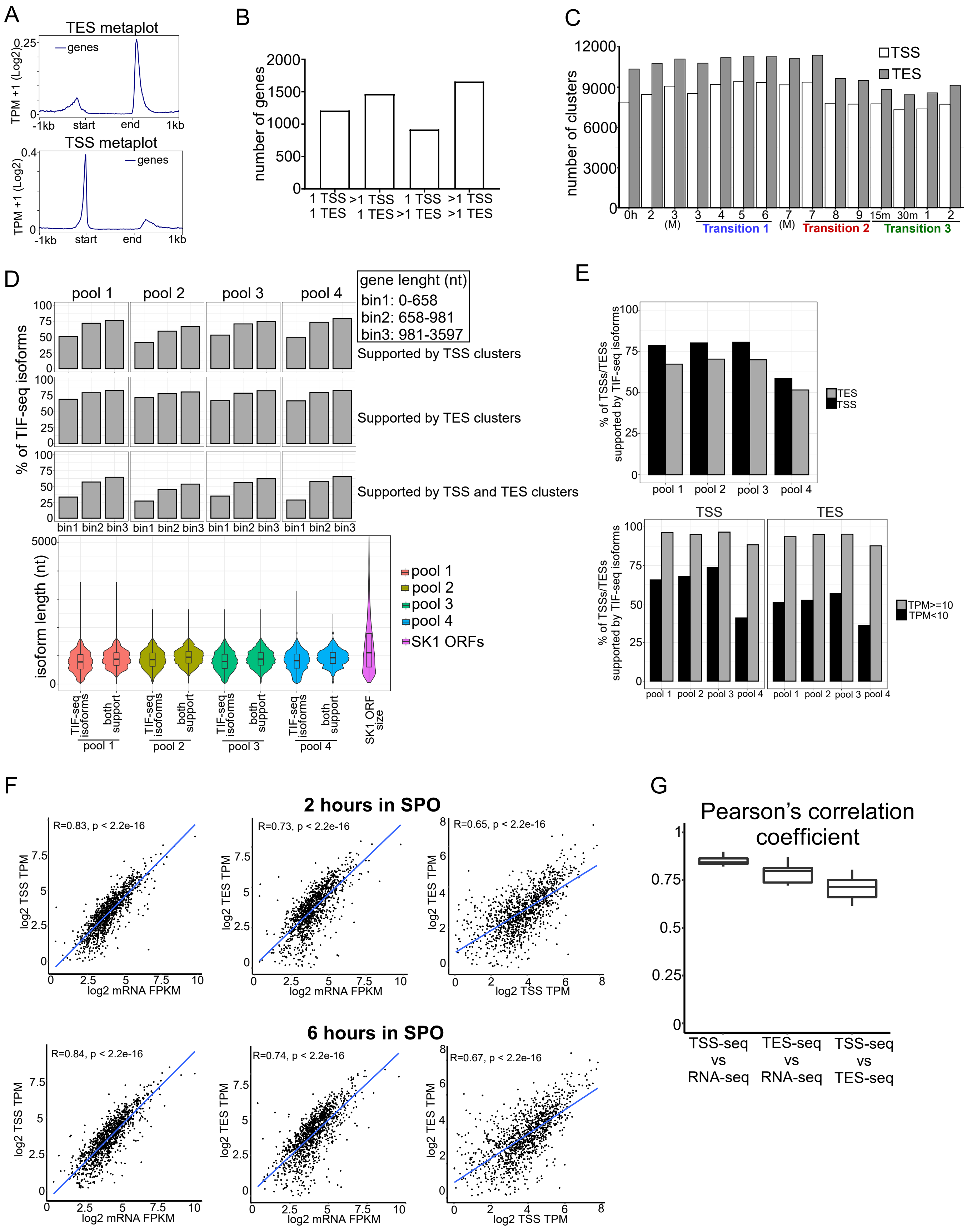

Fig. S1. Profiling of transcript heterogeneity during three synchronized cell fate transitions **a**. Meta-profiles of TSS and TES signals across the regions containing the ORF and 1 kb upstream/downstream. **b.** Number of genes with single or multiple TSSs and/or TES CAGEr clusters assigned to them. **c.** Numbers of predicted TSS and TES clusters in each time point during three cell fate transitions, T1, T2, and T3. The M stands for mock treated samples. **d.** Fractions of TIF-seq isoforms supported by TSS/TES clusters, separating the isoforms into three subsets by isoform length. Pool 1: samples collected from 0-2h SPO; pool 2: samples collected from 3-6h SPO; pool 3: samples collected from 7-9h SPO; pool 4: samples collected from 15, 30, 60 and 120 min after shift to YPD. At the bottom are length distributions of TIF-seq isoforms, in comparison to the length distribution of annotated ORFs. “Both support” means the considered isoforms are those supported by both TSS and TES clusters. **e.** Top: the fractions of TSS/TES clusters supported by TIF-seq isoforms. Bottom: the fractions of TSS/TES clusters supported by TIF-seq isoforms, separating the TSS/TES clusters into two subsets by expression level. **f.** Scatter plots displaying expression changes of TSS reads versus mRNA reads (top), TES reads against mRNA reads (middle) and TSS reads against TES reads (bottom). The Pearson correlation coefficients and p-values are also given. Data shown here were taken from 2 and 6 hours in SPO of three biological repeats. Data represents 1182 genes with only a single annotated TSS and TES, averaged from three biological repeats. **g.** Pearson’s correlation coefficients derived from plotting the relationship between the TPM of TSS-seq versus mRNA-seq (FPKM), TESs against mRNA, and TSSs against TESs described in f for all time points from the master time course.

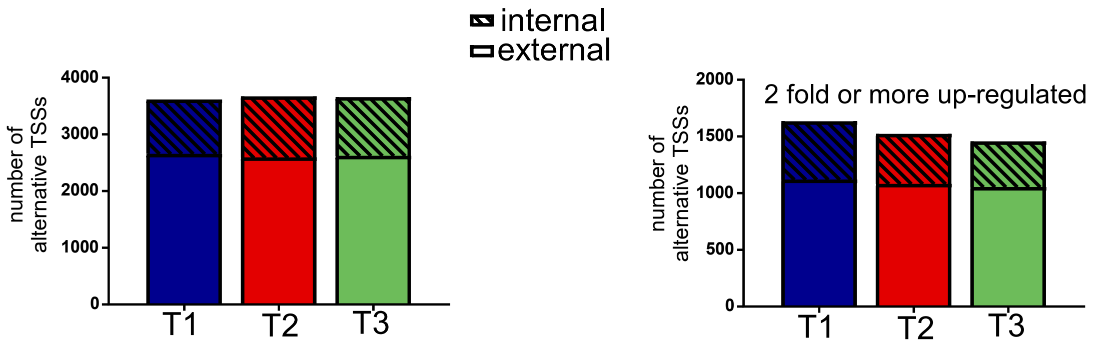

Fig. S2. Alternative TSSs and TESs are pervasive expressed during T1, T2 and T3
Barplots showing the numbers of external and internal alternative TSS clusters during each transition (left), as well as those which were at least two fold up-regulated (right). Alternative TSS clusters are classed as external if the feature is = 1kb upstream of an ORF running in the same orientation. Any other clusters in the same orientation are classed as internal. Alternative TSS clusters were included if the feature had at least 1 TPM of signal during at least 1 time point of the relevant transition.

Figure S3 (Chia et al)

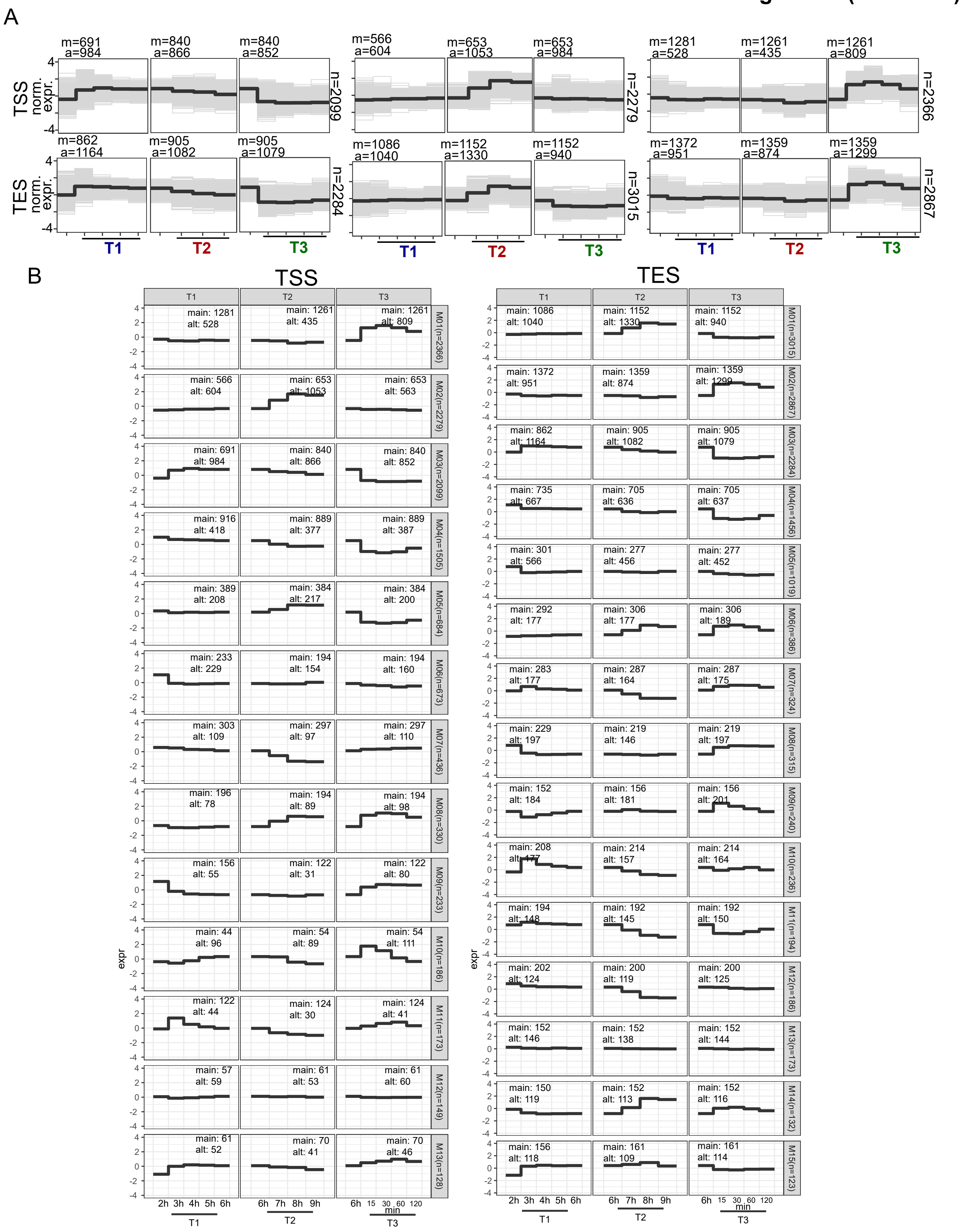

C

T1 main

Motif	p-values	% of Targets	% of Background	Best match
ATTAGCCGCCGA	1e-71	13.04%	1.33%	UME6
TCGTTACCCGTC	1e-56	23.52%	6.58%	STB2
AAACAAACAA	1e-21	14.67%	5.64%	HCM1

T1 alternative

Motif	p-values	% of Targets	% of Background	Best match
ATTAGCCGCC	1e-66	13.25%	1.68%	UME6
TTTTTTCCGC	1e-18	49.67%	35.14%	RGT1
CGCGCGGC	1e-17	30.24%	18.40%	RSC3

T2 main

Motif	p-values	% of Targets	% of Background	Best match
CGACACAAAA	1e-43	10.33%	1.13%	NDT80
GTCACCGCG	1e-30	36.10%	17.57%	MBP1
GTGTCAGAAA	1e-17	5.97%	1.04%	SUM1

T2 alternative

Motif	p-values	% of Targets	% of Background	Best match
CACAAAAA	1e-112	29.63%	6.51%	NDT80
TGTGCGGC	1e-22	24.17%	12.91%	STP1
AAAAAAAACCAC	1e-19	37.72%	24.92%	AZF1

T3 main

Motif	p-values	% of Targets	% of Background	Best match
GCGATGAGAT	1e-23	9.65%	2.30%	TOD6
AAAATTTTTCTC	1e-21	15.12%	5.61%	SFP1
ATGAGTCA	1e-20	13.17%	4.61%	GCN4

T3 alternative

Motif	p-values	% of Targets	% of Background	Best match
GCGATGAG	1e-149	22.07%	3.23%	TOD6
TTAAAAATTTTT	1e-127	25.68%	5.48%	SFP1
ATTTTTCACT	1e-69	28.83%	11.06%	STB3

D

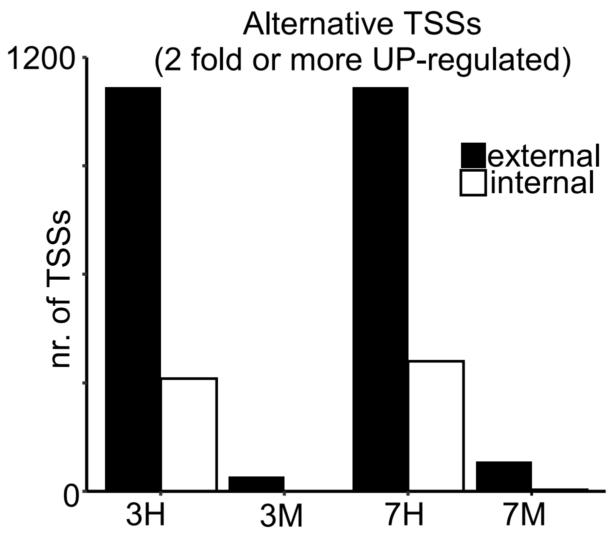

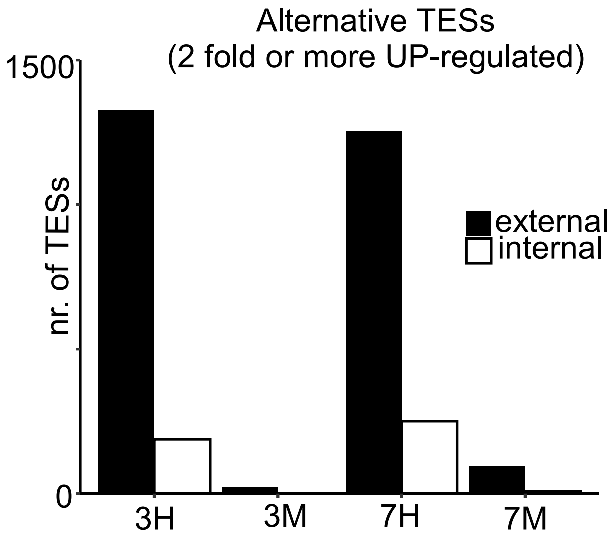

Fig. S3. Features of alternative TSSs

a. Top three co-expression modules for the TSS-seq and TES-seq time course, inferred by WGCNA analysis. The light-color curves represent the normalized expression levels of individual TSS/TES clusters, and the thick black curves depict the average expression levels within each module. Numbers (n) on the right-hand sides of the panels are the sizes of the modules. On top of panels are numbers of annotated main (m) and alternative (a) TSSs/TESs in each module for each transition. **b.** All co-expression modules (module size of >=100) for TSS (left) and TES (right) clusters, inferred by WGCNA. The black curves depict the average normalized expression levels within each module. Numbers on the right-hand sides of the panels are sizes of the modules. On top of panels are numbers of annotated main and alternative TSSs/TESs in each module for each transition. **c.** Top three enriched motifs predicted by HOMER in core promoters of genes in each of top three co-expression modules of TSSs, separating the main and alternative TSSs for analysis. The region [-150nt, 50nt] around the dominant TSS position was considered as the core promoter region. **d.** Increased expression of alternative TSSs/TESs during T1 and T2 depends induction of Ime1 and Ndt80, respectively. The number of alternative TSSs increased in usage (2-fold or more) were compared between 3H (T1, Ime1 induced) and 3M (mock treated), and between 7H (T2, Ndt80 induced) and 7M (mock treated).

Figure S4 (Chia et al)

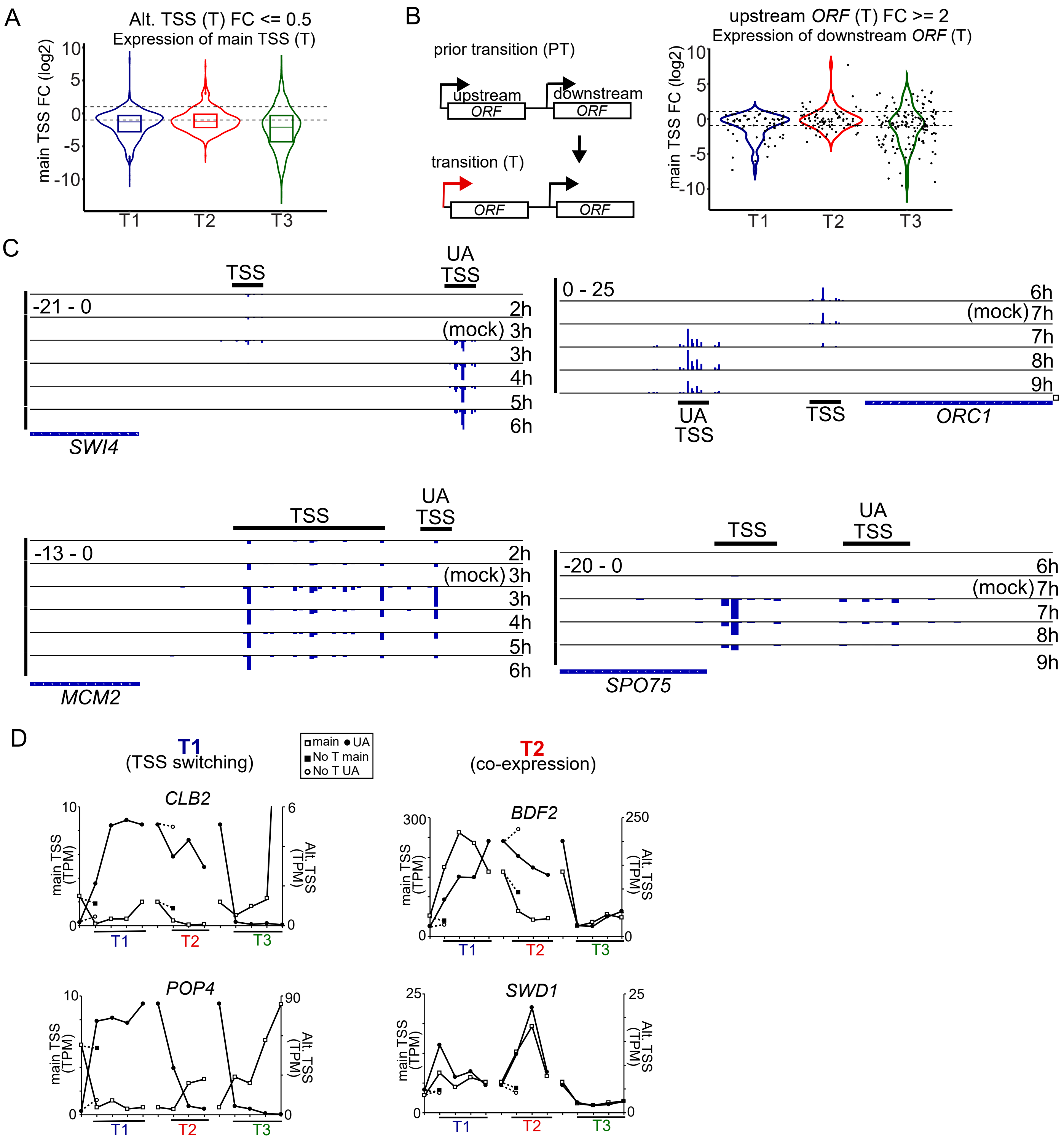

Fig. S4. Increased upstream alternative TSS usage has varying effects on expression from the main TSS

a. Decreased upstream transcription is frequently accompanied with decreased transcription from downstream promoters. The data in this figure were obtained from the TSS pairs whereby the alternative TSS was strictly upstream of both the main TSS of the same gene. Data were further subsetted to include only the time points whereby expression from the upstream alternative TSS was decreased by two fold or more. The log2 fold change of the downstream main TSS was plotted on the y axis. "T1" refers to transition 1 and represents the following comparisons: 3h, 4h, 5h or 6h vs 2h SPO. "T2" refers to transition 2 and represents the following comparisons: 7h, 8h or 9h vs 6h SPO. "T3" refers to transition 3 and represents the following comparisons: 15 min, 60 min or 120 min YPD vs 6h SPO. The horizontal dashed lines mark log2 fold changes of 1 or -1. The data represents TSS pairs from 516, 372 and 717 genes from T1, T2 and T3 respectively, and is an average of 3 biological repeats. **b.** Transcription of tandem gene pairs are mostly regulated independently. For this analysis, pairs of genes were selected if both members had the same orientation and if the two ORFs were < 200 base pairs apart. Gene pairs were further selected if the main TSS for the upstream member was upregulated by two-fold or more. The log2 fold change of the downstream main TSS was plotted on the y axis. "T1" refers to transition 1 and represents the following comparisons: 3h, 4h, 5h or 6h vs 2h SPO. "T2" refers to transition 2 and represents the following comparisons: 7h, 8h or 9h vs 6h SPO. "T3" refers to transition 3 and represents the following comparisons: 15 min, 60 min or 120 min YPD vs 6h SPO. The horizontal dashed lines mark log2 fold changes of 1 or -1. The data represents 23, 40 and 46 gene pairs from T1, T2 and T3 respectively, and is an average of 3 biological repeats. **c.** Examples of genes described in Fig 3e (*SWI4*, *ORC1*, *MCM2,* and *SPO75*) with multiple TSSs displayed in Integrative Genome Browser. Indicated are time points and TSS clusters (black horizontal lines). **d.** Main and alternative TSS expression changes at example loci during T1, T2, and T3. Negative controls representing mock treated samples for T1 and T2 (no induction of transition (T) for main TSS and upstream alternative TSS (UA)) were included. The y-axis represents TPM values of the main and alternative TSS. Graphs on the left depict examples of TSS switching events for *CLB2* and *POP4.* Graphs on the right depict examples of TSS co-expression for the genes *BDF2* and *SWD1.*

Figure S5 (Chia et al)

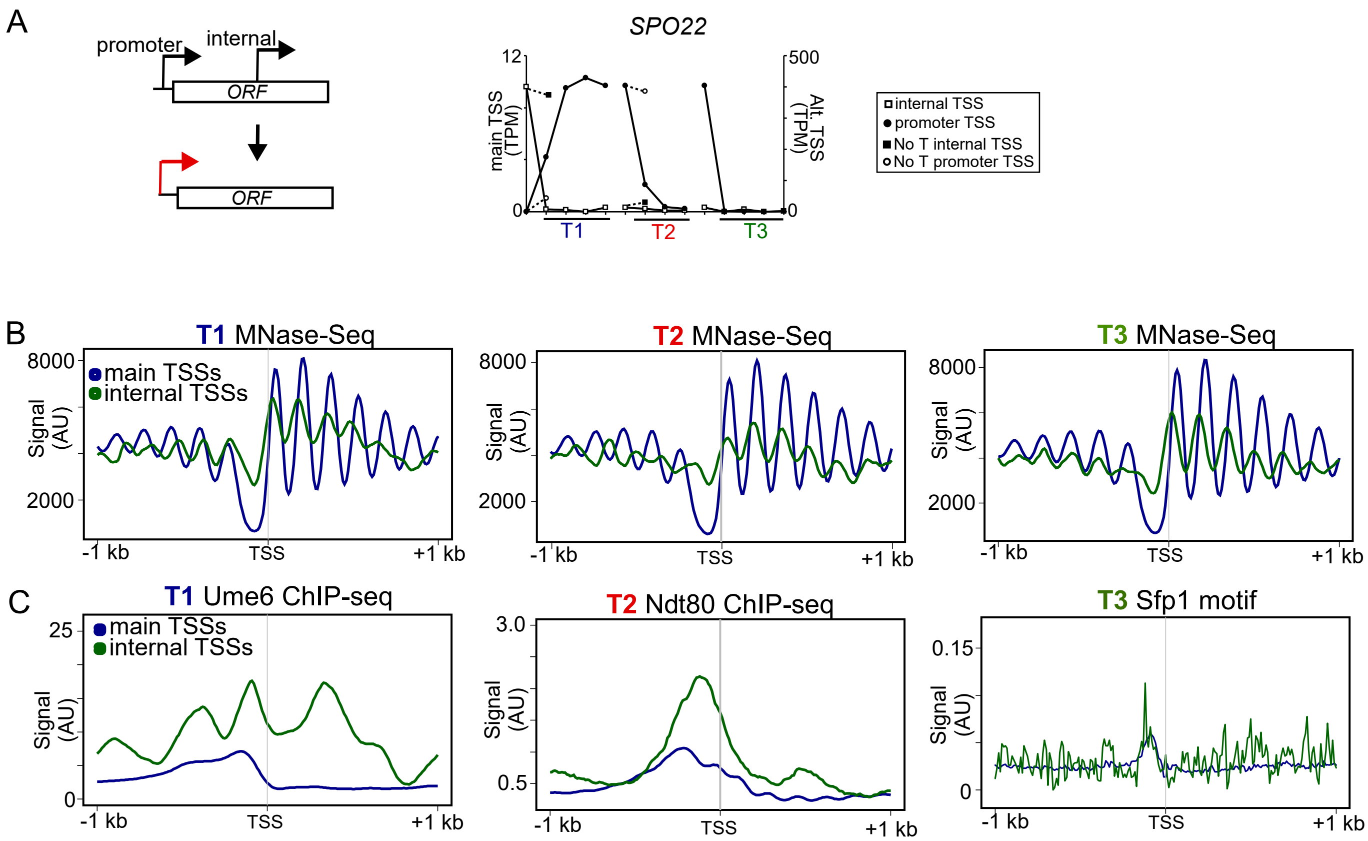

Fig. S5. Widespread dynamic regulation of alternative TSSs within ORFs during cell-fate transitions

a. Left: Some genes are dominantly expressed from an internal TSS and only switch to an upstream, external TSS during cell fate transitions. Right: Plot of the TPM signal of TSSs associated with *SPO22*, showing a stage specific shift from internal TSS usage to an upstream, external TSS. **b.** Meta-profiles of MNase-seq signals for main and internal TSSs in each transition. **c.** Meta-profiles of Ume6 ChIP-seq signals for main and internal TSSs in T1 (left), Ndt80 ChIP-seq signals in T2 (middle) and Sfp1 motif density in T3 (right).

Figure S6 (Chia et al)

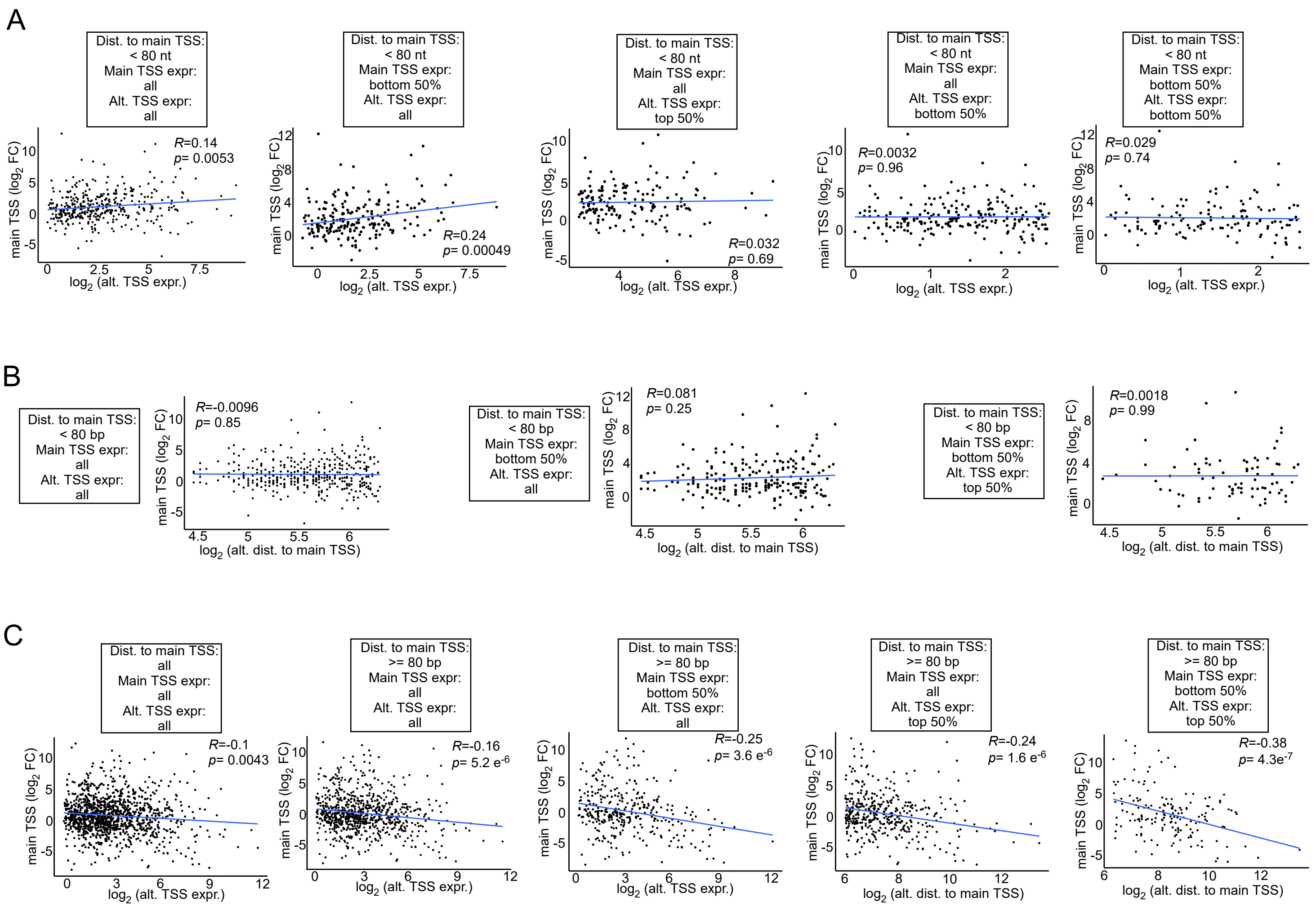

Fig. S6. Features explaining the response of main expression changes upon increased alternative TSS usage

a. Scatter plots of main TSS expression changes (transition versus prior transition state, log2, fold change) against alternative TSS expression values during cell fate transition (log2, TPM). The data presented is combined for T1 (6h vs 2h in SPO), T2 (8h vs 6h in SPO), and T3 (60 min YPD vs 6h SPO). The Pearson correlation coefficient and its p-value are displayed. Depicted in the box are cut-offs used for subsetting of data points. **b.** Similar type of scatter plots as a, except that the main TSS expression changes (transition versus prior transition state, log2, fold change) against distances between the main and alternative TSSs (log2, nucleotides) are shown. **c.** Similar type of scatter plots as displayed and a and b, except that different subsetting of the data points are shown.

Figure S7 (Chia et al)

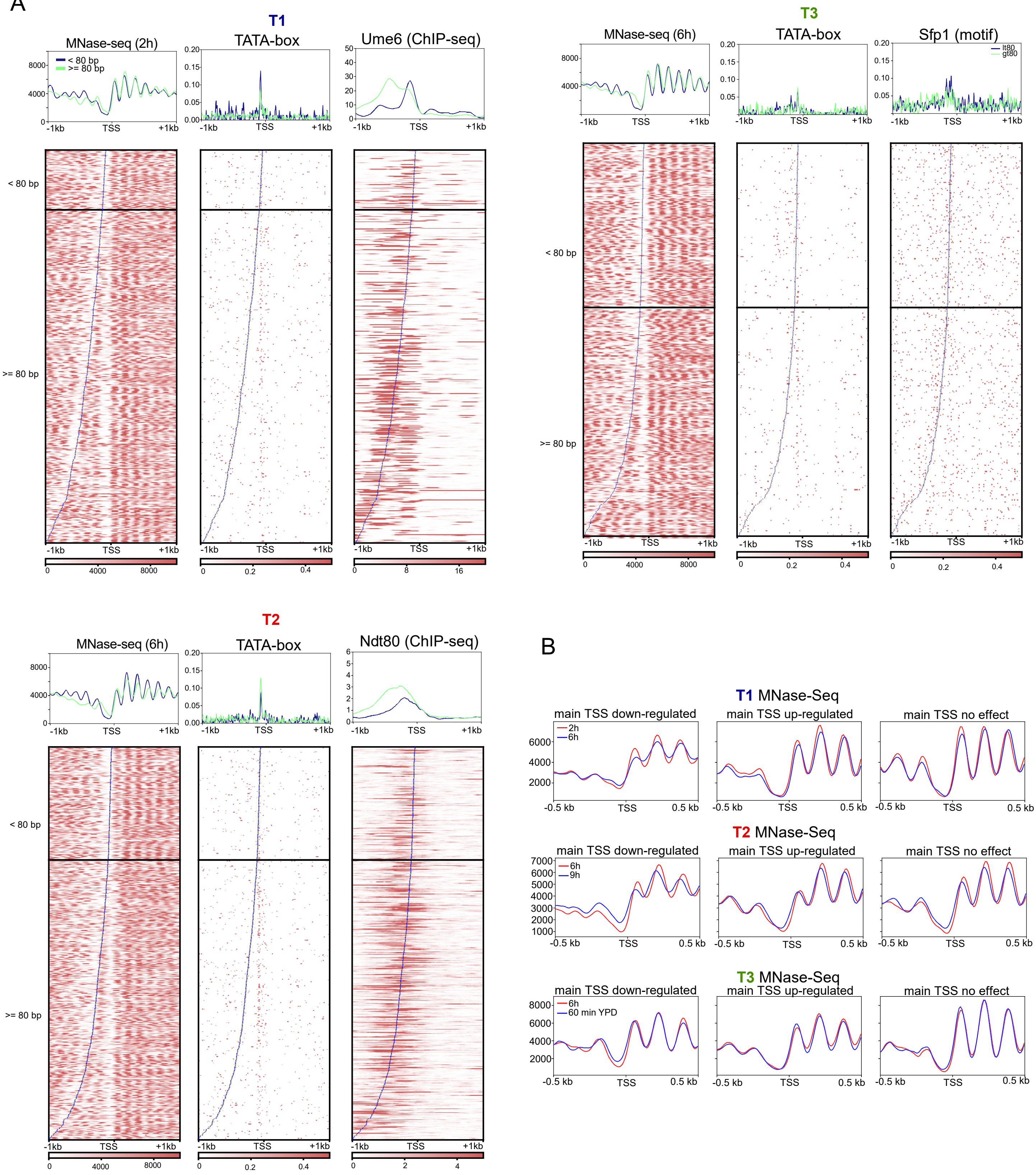

Fig. S7. Metagene analyses of genes with upstream alternative TSSs

a. Meta-profiles of genes whereby the distance between main and upstream alternative TSS is less than 80 bp (< 80bp) or more than 80 bp (>= 80bp). Signals were centred on the main TSS. Displayed are MNase-seq signals (T1, T2 and T3), ChIP-seq signal for Ume6 (T1) and Ntd80 (T2), and motif Sfp1 density (T3), and TATA box motif density (T1, T2 and T3). The Ndt80 Chip-seq data were obtained from (Nocedal et al 2017). Figures were generated by deepTools. **b.** Meta-profiles of MNase-seq signals, centered on the main TSS, in genes whereby levels of an upstream alternative TSS was upregulated by two fold or more by DESeq2. The downregulated category represents genes whereby expression from the main TSS also decreases by two-fold or more. The upregulated category represents genes whereby expression from the main TSS increases by two-fold or more. The no effect category represents genes whereby expression from the main TSS does not change appreciably (two-fold in either direction), or the fold changes were not statistically significant by DESeq2 (FDR < 0.05).

Figure S8 (Chia et al)

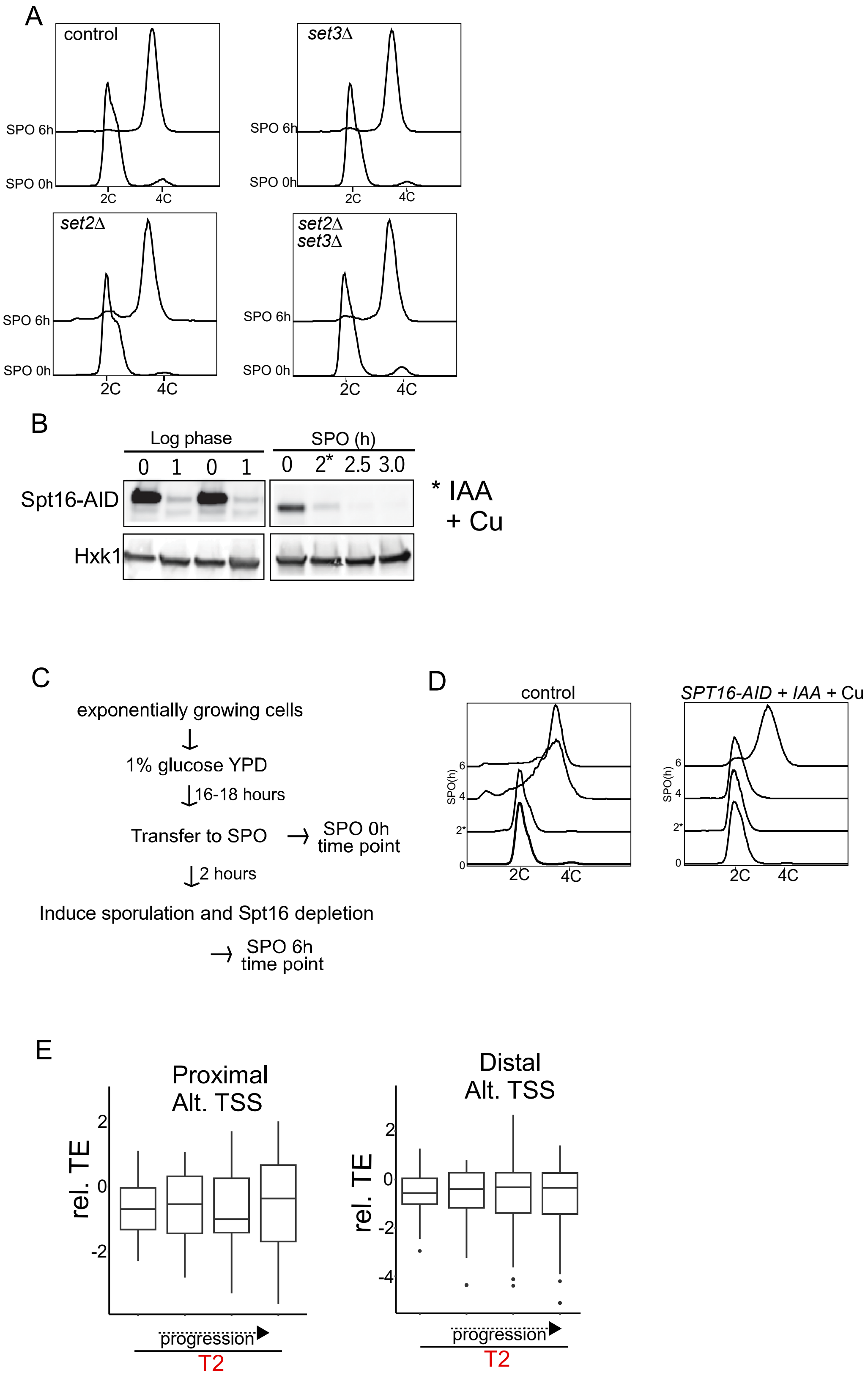

Fig. S8. Chromatin factors mediate repression exercised by upstream alternative TSSs and genes expressing upstream alternative TSSs display a wide range of translational efficiencies

a. Flow cytometry analysis of DNA content of control (FW2795), *set3Δ* (FW5770), *set2Δ* (FW5767) and *set2Δset3Δ* cells (FW2912). All these cells also had the *pCUP-IME1* and *GAL4.ER pGAL-NDT80* alleles. Samples were taken at indicated time points, fixed, and DNA content was measured by propidium iodide staining; 50 μM CuSO4 was added 2h after the cells were transferred to SPO (*). At least 50,000 cells were analyzed at each time point. Results are representative of at least three independent repeats. **b.** Western blot showing depletion of Spt16-AID protein in log phase (left) and when cells shifted to SPO medium (right). Cells used here had the *SPT16-AID*, *pCUP-TIR1*, *pCUP-IME1* and *GAL4.ER pGAL-NDT80* alleles (FW6083). For the SPO time points, expression of *IME1* and degradation of tagged Spt16 were induced at the same time at 2 h (*) by the addition of CuSO4 (50 μM) and IAA (500 μM). Results are representative of two independent repeats. **c.** Flowchart for the time course with mutant and control cells. Diploid cells were grown to saturation in YPD and collected for the 0h time point. Remaining cells were then transferred to SPO and IME1 was induced 2h later. By 6h in SPO, cells were arrested in meiotic prophase and were collected for another time point. **d.** Flow cytometry analysis of DNA content of control (FW6109) and *SPT16-AID* cells (FW6083). All these cells harboured the *SPT16-AID*, *pCUP-IME1* and *GAL4.ER pGAL-NDT80* alleles. Control cells lacked the *pCUP-TIR1* gene. Samples were taken at indicated time points, fixed, and DNA content was measured by propidium iodide staining; CuSO4 (50 μM) and IAA (500 μM) were added 2h after the cells were transferred to SPO (*). At least 50,000 cells were analyzed at each time point. Results are representative of at least three independent repeats. f. Box plots of translation efficiency values obtained from (Brar et al. 2012) for the subset of relaxed switching genes identified in transition 2 as described in Fig. 3c. Left: Genes showing TSS switching in T2 under the relaxed criteria are included, and subsetted for tandem TSSs proximal to each other (< 80 bp). Right: genes subsetted for tandem TSSs distal to each other (= 80 bp).
