# Supplementary material for: High-resolution analysis of cell-state transitions in yeast suggests widespread transcriptional tuning by alternative starts
Source: Genome Biol. 2021 Jan 14;22:34. doi: 10.1186/s13059-020-02245-3 (PMC7807719; doi:10.1186/s13059-020-02245-3)
Supplement: Supplementary file 7 — Additional file 7: Table S1. Yeast strain genotypes. [file 13059_2020_2245_MOESM7_ESM.docx]

## Table S1. Yeast strain genotypes

| **Strain** | **Genotype** |
| --- | --- |
| FW1208 | *MAT***a,** *ho::LYS2, ura3, leu2::hisG, his3::hisG, trp1::hisG, UME6-3V5::His3MX*  *MATα***,** *ho::LYS2, ura3, leu2::hisG, his3::hisG, trp1::hisG, UME6-3V5::His3MX* |
| FW1509 | *MAT****a****, ho::LYS2, lys2, ura3, leu2::hisG, his3::hisG, trp1::hisG* |
| FW1510 | *MATα, ho::LYS2, lys2, ura3, leu2::hisG, his3::hisG, trp1::hisG* |
| FW1511 | *MAT****a****, ho::LYS2, lys2, ura3, leu2::hisG, his3::hisG, trp1::hisG*  *MATα, ho::LYS2, lys2, ura3, leu2::hisG, his3::hisG, trp1::hisG* |
| FW2795 | *MAT****a****, ho::LYS2, lys2, ura3, leu2::hisG, his3::hisG, trp1::hisG,*  *irt1::pCUP-3HA-IME1::HphMX , ndt80::pGAL-NDT80::TRP1, ura3::pGPD1-*  *GAL4(848).ER::URA3*  *MATα, ho::LYS2, lys2, ura3, leu2::hisG, his3::hisG, trp1::hisG,*  *irt1::pCUP-3HA-IME1::HphMX , ndt80::pGAL-NDT80::TRP1, ura3::pGPD1-*  *GAL4(848).ER::URA3* |
| FW2912 | *MAT****a****, ho::LYS2, lys2, ura3, leu2::hisG, his3::hisG, trp1::hisG,*  *irt1::pCUP-3HA-IME1::HphMX , ndt80::pGAL-NDT80::TRP1, ura3::pGPD1-*  *GAL4(848).ER::URA3, set3::His3MX, set2::His3MX*  *MATα, ho::LYS2, lys2, ura3, leu2::hisG, his3::hisG, trp1::hisG,*  *irt1::pCUP-3HA-IME1::HphMX , ndt80::pGAL-NDT80::TRP1, ura3::pGPD1-*  *GAL4(848).ER::URA3, set3::His3MX, set2::His3MX* |
| FW5767 | *MAT****a****, ho::LYS2, lys2, ura3, leu2::hisG, his3::hisG, trp1::hisG,*  *irt1::pCUP-3HA-IME1::HphMX , ndt80::pGAL-NDT80::TRP1, ura3::pGPD1-*  *GAL4(848).ER::URA3, set2::His3MX*  *MATα, ho::LYS2, lys2, ura3, leu2::hisG, his3::hisG, trp1::hisG,*  *irt1::pCUP-3HA-IME1::HphMX , ndt80::pGAL-NDT80::TRP1, ura3::pGPD1-*  *GAL4(848).ER::URA3, set2::His3MX* |
| FW5770 | *MAT****a****, ho::LYS2, lys2, ura3, leu2::hisG, his3::hisG, trp1::hisG,*  *irt1::pCUP-3HA-IME1::HphMX , ndt80::pGAL-NDT80::TRP1, ura3::pGPD1-*  *GAL4(848).ER::URA3, set3::His3MX*  *MATα, ho::LYS2, lys2, ura3, leu2::hisG, his3::hisG, trp1::hisG,*  *irt1::pCUP-3HA-IME1::HphMX , ndt80::pGAL-NDT80::TRP1, ura3::pGPD1-*  *GAL4(848).ER::URA3, set3::His3MX* |
| FW6083 | *MAT****a****, ho::LYS2, lys2, ura3, leu2::hisG, trp1::hisG,*  *irt1::pCUP-3HA-IME1::HphMX , ndt80::pGAL-NDT80::TRP1, ura3::pGPD1-*  *GAL4(848).ER::URA3, SPT16::SPT16-3V5-AID::KanMX6, his3::pCUP-OsTIR::His3MX*  *MATα, ho::LYS2, lys2, ura3, leu2::hisG, his3::hisG, trp1::hisG,*  *irt1::pCUP-3HA-IME1::HphMX , ndt80::pGAL-NDT80::TRP1, ura3::pGPD1-*  *GAL4(848).ER::URA3, SPT16::SPT16-3V5-AID::KanMX6, his3::pCUP-OsTIR::His3MX* |
| FW6109 | *MAT****a****, ho::LYS2, lys2, ura3, leu2::hisG, his3::hisG, trp1::hisG,*  *irt1::pCUP-3HA-IME1::HphMX , ndt80::pGAL-NDT80::TRP1, ura3::pGPD1-*  *GAL4(848).ER::URA3, SPT16::SPT16-3V5-AID::KanMX6*  *MATα, ho::LYS2, lys2, ura3, leu2::hisG, his3::hisG, trp1::hisG,*  *irt1::pCUP-3HA-IME1::HphMX , ndt80::pGAL-NDT80::TRP1, ura3::pGPD1-*  *GAL4(848).ER::URA3, SPT16::SPT16-3V5-AID::KanMX6* |
