# Supplementary material for: High-resolution analysis of cell-state transitions in yeast suggests widespread transcriptional tuning by alternative starts
Source: Genome Biol. 2021 Jan 14;22:34. doi: 10.1186/s13059-020-02245-3 (PMC7807719; doi:10.1186/s13059-020-02245-3)
Supplement: Supplementary file 9 — Additional file 9. Review history. [file 13059_2020_2245_MOESM9_ESM.docx]

Review History

**First round of review**

**Reviewer 1**

**Are you able to assess all statistics in the manuscript, including the appropriateness of statistical tests used?**

There are no statistics in the manuscript.

**Comments to author:**

This manuscript includes a group of global expression measurements for two stages of gametogenesis, and entry into budding, in budding yeast. These measurements aim to measure both transcript ends, transcript levels, and nucleosome positioning for each state examined. The authors generated data here that will be useful for the field. They use the data to make several conclusions, including that many transcript isoforms are seen in different conditions, that some isoforms show a relationship in their regulation to main transcripts, and that chromatin modifiers impact these patterns. Some of these conclusions are very well supported and others are not, which is understandable given the very large number of conclusions here. But the paper would be stronger if it either focused on the conclusions that are well supported by their data or added additional clarification or support for those that are not.

There are four major concerns with the data that limit its applicability to some of the authors conclusions:

1. It is not established here that the TSS seq and TES seq are quantitative. The authors analyze their data as though the peak clusters are linearly quantitative but from examining the authors references for these methods, I found no calibration showing that peak height is linearly quantitative with amount of transcription and it is known that sequencing read outs from peaks are prone to artifacts from library preparation biases. My reading of the existing literature cited is that these methods allow identification of isoforms but may not allow linearly quantitative analyses of the type employed in several places here. This is a particular concern given that many analyses show poor signal or unexpected mixed results. This is what one would expect to see see when noise is dominating one's analyses.

2. Possibly related to 1, the quantitative analyses employed in some places are not convincing. For example, the authors claim that their replicates "correlated remarkably" (p8) but the R squared is only 0.6. This is poor for most genomic expression measurements in yeast. The cluster analysis shown is also not so good. In the TSS data for example it seems that 4A is more similar to 5A and 6A than 4B and 4C which are more similar to some 6 h samples. Why is this? And then how can the authors quantitatively compare values over time? On page 8, they apply a computation method to identify clusters because there are too many peaks but do not describe this sufficiently and also do not explain how they are confident that this is a fair analysis. Only 50% of TSS sites match TIF sites but the explanation is not sufficient. If it is an issue of insufficient sequencing, would it not be solved by more sequencing? These issues and those in part 1 undermine many of the quantitative claims in the manuscript, such as those related to the temporal relationship between different isoforms.

3. The language is vague or sloppy in many places in a way that causes misleading statements. For example, the authors state that their data cannot show causality but also repeatedly say that one transcript change causes an effect on another isoform. They mix words that refer to transcription with those that refer to transcripts. The first sentence, for example, is not correct. The ends of messenger RNAs are the UTR sequences, not the TSS and TES, which are DNA sequences. This is mentioned because the authors do make conclusions about transcription without direct measurements of transcription. For example, many statements are made about what the levels of transcript isoforms say about transcription co-regulation or repression or switching but they have no evidence that the isoforms are of similar transcript stability.

4. The authors focus on the relationship between different isoforms that are produced by transcription factors but they are manipulating the levels of transcription factors to do these experiments. This certainly affects natural transcriptional regulation. This may not itself be a problem in identifying isoforms but it may be a problem in their quantitative analysis. This must be acknowledged, at minimum, as a major caveat of quantitative analyses of isoforms.

So where does this leave us? The data are valuable but not validated sufficiently for reproducibility and applicability to the quantitative analyses applied later.. The chromatin data, both MNASE and comparison in Set mutants, may allow the authors to find qualitative answers about relationships between isoforms that they wish to include without requiring them to over interpret peak values in TSS and TES data. The manuscript would likely improve by focusing on a few strong statements that can be proven.

Minor points:

1.The authors do not sufficiently recognize the work of Zhang and Neiman. This should be cited in additional places because it first showed that Ndt80 causes expression of transcript isoforms from within genes in gametogenesis.

2.The title of the manuscript does not fit with the data in it. There is no mention of transcriptional tuning anywhere outside the title and abstract and intro but rather the data shows that there are a variety of relationships including no relationship between isoforms. Tuning means changes in levels that are controlled by a mechanism and the authors do not show any causality in alternative start use and changes in levels of transcription.

3. I did not understand the description of the analyses in the switching section. It seems that the rules described are for identifying instances of replacement of one isoform with another but co-existence and changes in levels could also occur. To measure tuning or switching as the title implies as a goal, one would expect an analysis of temporal patterns not fold changes or expression levels. Why not use these same analyses as were used to globally assess relationships between isoforms? Why does the switching analysis use arbitrary cut offs that require certain fold changes and expression levels? My attempt to understand these categories based on the cited papers in this section did not clarify these choices. Are the authors solely looking for cases that have all the same quantitative features as NDC80 does in these new data?

4. Some sentences are too vague to be helpful, including "We propose that TSS usage changes drive a widespread dynamic transcriptional program, impacting mRNA and protein production." And "Our data suggest that increased alternative upstream TSS usage can have a range of outcomes on transcription from the main TSS, and upstream long transcript isoforms possess different translation efficiencies."

5. The discussion ends by saying the data are consistent with previous observations and then simply citing two of the authors recent papers. It would be helpful to discuss how the data fit in the field more broadly.

**Reviewer 2**

**Are you able to assess all statistics in the manuscript, including the appropriateness of statistical tests used?**

No, I do not feel adequately qualified to assess the statistics.

**Comments to author:**

Review:

This publication by the Van Werven lab aims at describing the changes in TSSs (and TESs) at a genome-wide level during cell-state transitions in S. cerevisiae. To achieve this, the authors use several technics such as TSS-seq, TES-seq, mRNA-seq, TIF-seq and MNase-seq at several key time points during meiosis but also growth in rich medium. The take-home message of the publication is that TSS positioning is highly dynamic during cell-phase transitions and can lead to several outcomes of gene expression. An increase in Upstream Alternative TSSs (UA TSS) leads to either downstream gene repression or activation. This difference appears to mainly be set by the distance between the UA TSS and the original TSS. The authors also describe the importance of chromatin-related factors (Set2, Set3 and Spt16) in the regulation of the original TSS.

This publication is quite amazing in terms of genome-wide data generated and subsequent analyses. However, this might be the weakness of the publication as well. Many different criteria are taken into account for the analyses and this is perhaps why this article lacks a certain homogeneity. Genome Biology appears as a good target for this publication but I would suggest some more experiments, precisions or changes to (try to) improve this work. The comments below should help to make it more accessible to the reader and suitable for publication.

My main comment is that the molecular bases of TSS switching are not directly addressed. Recent work from Friedman lab (Klein-Brill et al., 2019), Shore and Libri labs (Challal et al., 2018; Kubik et al., 2019) and your lab (Wu et al., 2018) have shown the importance of both transcription factors and nucleosome positioning in TSS regulation.

- Are UA TSS related to new (upstream) or extended NDRs? Does the distance between UA TSS and main TSS influence chromatin structure? MNase-seq data are presented in the supplementary figures only for the internal TSS and main TSS but not for UA TSS. For example, when the distance is <80bp, one might expect that NDRs are not changed whereas a new NDR might appear when the distance is >80bp.

- The distance between the TATA-Binding Site (TBS) and +1 nucleosome is important for transcription initiation (Kubik et al., 2018). In cases of an UA TSS, is transcription initiation related to another TBS? Same or different TBS for Alternative proximal and Alternative distal classes?

- Are Ume1 and Ndt80 recruited in the vicinity of UA TSSs? This seems true for the internal TSS but not shown for UA TSS.

Some more specific comments:

- If understood correctly, Figure 2B says that about 3000 genes have Alternative TSS clusters. However, Figure 1F says that around roughly 2000 genes have more than 1 TSS cluster. Where is this discrepancy coming from?

- In Figure 2C, the Alt TSS taken into account are the ones that show more than 2 fold increase. So, they are not the same as in Figure 1B for which definition is less strict. Globally, for all the publication, it would be of interest to indicate how many genes are taken into account directly on the graphs (at the top of histograms for example).

- Figure 3C: It seems that relaxed/moderate/strict criteria complexifies the analysis without bringing important novel conclusions.

- Figure 3E: It might be good to add a snapshot of an example of TSS switching and TSS co-reg, at least as a supplementary figure.

- Figure 5 is of big interest for the conclusion of the publication. In my experience, trying to find a linear correlation with a good correlation coefficient does not work well (biological responses are not linear most of the time). It is just a suggestion but what about trying to represent the same data with histograms? An example with Figure 5A. Genes might be separated into 2 groups (<80bp and >80bp) and the main TSS fold-change might be represented according to these 2 groups. It will simplify the overall design and will stress what you propose in the model.

- Figure 5B: a correlation of -0.1 (even with a good p-value) is not satisfying.

- Figure 6E: the metagene analysis is presented without statistics; it is therefore difficult to conclude that chromatin structure is really different.

- Figure 7A: the data are presented with the relaxed criteria. Moderate and strict classes could also be considered. It might increase the statistical power of the analysis.

- Figure 7C: some of the cases are not well proven in the publication. For example, I agree that for the distal alternative TSS usage, the case at the bottom is shown in the publication (Strong>Weak>TSS switching> decrease) and well demonstrated. But, for many "protein levels", there is no obvious information in the publication and there should therefore be a question mark. Not sure for example that Proximal>Strong>Weak >co-upreg is really shown in the publication.

- There is the possibility that you underestimate the Alternative TSSs isoforms since these transcripts might be discarded by premature termination (Nrd1-Nab3-Sen1 pathway) or NMD. Some isoforms might be transcribed but rapidly degraded giving a low isoform signal while having an impact on the downstream main TSS through chromatin regulation. It is probably really complicated to reveal such a phenomenon but would be nice to discuss it.

**Authors Response**

**Point-by-point responses to the reviewers’ comments:**

Reviewer #1:

This manuscript includes a group of global expression measurements for two stages of gametogenesis, and entry into budding, in budding yeast. These measurements aim to measure both transcript ends, transcript levels, and nucleosome positioning for each state examined. The authors generated data here that will be useful for the field. They use the data to make several conclusions, including that many transcript isoforms are seen in different conditions, that some isoforms show a relationship in their regulation to main transcripts, and that chromatin modifiers impact these patterns. Some of these conclusions are very well supported and others are not, which is understandable given the very large number of conclusions here. But the paper would be stronger if it either focused on the conclusions that are well supported by their data or added additional clarification or support for those that are not.

There are four major concerns with the data that limit its applicability to some of the authors conclusions:

1. It is not established here that the TSS seq and TES seq are quantitative. The authors analyze their data as though the peak clusters are linearly quantitative but from examining the authors references for these methods, I found no calibration showing that peak height is linearly quantitative with amount of transcription and it is known that sequencing read outs from peaks are prone to artifacts from library preparation biases. My reading of the existing literature cited is that these methods allow identification of isoforms but may not allow linearly quantitative analyses of the type employed in several places here. This is a particular concern given that many analyses show poor signal or unexpected mixed results. This is what one would expect to see see when noise is dominating one's analyses.

*We agree with the reviewer that it is important to thoroughly assess the quantitative behaviour of the TSS-seq signals. It is worth noting that the TSS seq protocol used in this study has been adapted from previously published work (Arribere and Gilbert, 2013) and has been widely in different genome wide studies to quantitatively measure levels of capped RNAs in yeast, from studies as recent as 2018 (https://www.ncbi.nlm.nih.gov/pmc/articles/PMC6239972/).*

*We have now included a comprehensive analysis of the comparisons between RNA-seq, TSS-seq, and TES-seq (Additional files 1-4). We have included genes of which we identified as having only one TSS or TES. We have calculated all the pairwise correlation metrics between TSS-seq, TES-seq and mRNA-seq. These data demonstrate that TSS-seq and RNA-seq signals correlate well. We found medium correlation of 0.83 across all time points (Additional file 1: Figure s1f and S1g and Additional file 3). A correlation of ~0.8 to 0.9 between TSS-seq and mRNA-seq is consistent with other TSS sequencing techniques described as “robust” in other papers (https://www.ncbi.nlm.nih.gov/pmc/articles/PMC6075671/, https://genome.cshlp.org/content/early/2020/07/02/gr.261545.120) which is line with what others have reported.*

*It is also worth noting that the RNA-seq and TSS-seq were performed on the same sample, but not the same RNA species. While the TSS-seq relies on the presence of capped-RNA and polyA site, RNA-seq depends on the presence of a polyA site only. Hence RNA-seq also detects uncapped mRNAs that are in the process of being degraded. This can, at least in part, explain why there is a difference between the two types of procedures. Indeed, genes with signals on the lower end of the graph show a stronger signal for RNA-seq (Additional file 1: Figs S1e and S1f and Additional files 3). Additionally, for some instances the TSSs may be fuzzy, which can make it challenging to capture the true TSS-signal levels. Finally, the library preparation protocols between TSS-seq and RNA-seq differ quite a bit. To control for detection of “artificial” signals from the TSS-seq we used a so-called non-decapping control to identify and remove these spurious peaks.*

*We also plotted the RNA-seq and TES-seq comparisons of all time points (Additional file 1: Figs S1f and S1g and Additional file 4). It is worth noting the TES-seq and RNA-seq correlated less well with a mean correlation of 0.75. Some of these differences may be explained by the difference in RNA species. The TES-seq was performed on fragmented polyA purified RNA, while RNA-seq was performed on total RNA. This may explain why some of the weakly expressed RNAs are less detected by this method.*

*The correlation between TSS-seq and TES-seq is perhaps unsurprisingly the least good. Biological differences (capped versus non-capped), and sample preparation (library protocol) can account for the differences between TSS-seq and TES-seq. Nevertheless, since the TSS-seq vs RNA-seq / TES-seq vs RNA-seq signals generally correlated well (except for the very weakly expressed genes), we are confident to use the TSS-seq and TES-seq data to make quantitative statements.*

2. Possibly related to 1, the quantitative analyses employed in some places are not convincing. For example, the authors claim that their replicates "correlated remarkably" (p8) but the R squared is only 0.6. This is poor for most genomic expression measurements in yeast. The cluster analysis shown is also not so good. In the TSS data for example it seems that 4A is more similar to 5A and 6A than 4B and 4C which are more similar to some 6 h samples. Why is this? And then how can the authors quantitatively compare values over time? On page 8, they apply a computation method to identify clusters because there are too many peaks but do not describe this sufficiently and also do not explain how they are confident that this is a fair analysis. Only 50% of TSS sites match TIF sites but the explanation is not sufficient. If it is an issue of insufficient sequencing, would it not be solved by more sequencing? These issues and those in part 1 undermine many of the quantitative claims in the manuscript, such as those related to the temporal relationship between different isoforms.

*We thank the reviewer for pointing this out. We had a closer look at the correlations between samples and repeats. We have now included all correlation data for all the time points and replicates (Additional file 2). There are two conclusions that we can extract from these data. First, the biological replicates correlated extremely well (0.95 to 1). The second point is that indeed some time points closely correlated with each other. This can be explained by the fact they belong to the same transition. For example, the time point 3, 4, 5 and 6 do not differ much in terms of global expression changes from each other because they belong to the T1 transition.*

*Regarding the TIF-seq difference between TSS-seq and TES-eq, we agree that in an ideal world the datasets should be overlapping perfectly. The TIF-seq technique has its limitations however (https://www.ncbi.nlm.nih.gov/pmc/articles/PMC4111111/). First, TIF-seq does not give quantitative information of mRNA isoform usage. Second, TIF-seq has a strong bias towards short transcripts and may therefore amplify weakly expressed short transcripts. This is also clear in our own dataset (Additional file 1: fig S1d). Despite the bias towards shorter transcripts, we see that the longer transcripts (which are more representative for coding genes) show a better overlap (more than 75%) (Additional file 1: fig S1d). Possibly, the shorter transcripts detected in TIF-seq are weakly expressed transcripts and therefore remain undetected in our TSS-seq procedure. We think that is beyond the scope of the manuscript to further sequence the TIF libraries in order to obtain a better overlap.*

3. The language is vague or sloppy in many places in a way that causes misleading statements. For example, the authors state that their data cannot show causality but also repeatedly say that one transcript change causes an effect on another isoform. They mix words that refer to transcription with those that refer to transcripts. The first sentence, for example, is not correct. The ends of messenger RNAs are the UTR sequences, not the TSS and TES, which are DNA sequences. This is mentioned because the authors do make conclusions about transcription without direct measurements of transcription. For example, many statements are made about what the levels of transcript isoforms say about transcription co-regulation or repression or switching but they have no evidence that the isoforms are of similar transcript stability.

*We thank you reviewer for pointing this out. We have fixed the statements regarding the inaccurate use of the word “transcription” throughout the manuscripts. Regarding TSS and TES, have defined these as transcript start site and transcript end site, and not transcription start site and transcription end site in our manuscript. We agree that TSS and TES refer often to transcription, but we were careful to define as transcript start site and transcript end site. It is worth noting that these terms are often used interchangeably. For example, TSS-seq technique is referred to as transcription start-site sequencing, while in reality this technique maps the start of the transcript and not the exact place where RNA polymerase II is loaded.*

4. The authors focus on the relationship between different isoforms that are produced by transcription factors but they are manipulating the levels of transcription factors to do these experiments. This certainly affects natural transcriptional regulation. This may not itself be a problem in identifying isoforms but it may be a problem in their quantitative analysis. This must be acknowledged, at minimum, as a major caveat of quantitative analyses of isoforms.

*The reviewer correctly points out that for T1 and T2 we use the synchronization system by manipulating the expression of the transcription factors Ime1 and Ndt80 respectively. The expression from heterologous promoter of Ime1 (CUP1-IME1) and Ndt80 (GAL-NTD80) could lead to mis-expression of transcripts. In part, we agree with this. However, in the case of Ime1 we think this is unlikely because of several reasons. First, CUP1-IME1 expression is not much different from when IME1 expressed is from its endogenous promoter, which is also a strong promoter. More importantly Ime1 does not act on its own in activating the genes in T1. Ime1 needs to interact with Ume6 to activate the transcripts in T1. Third, cells give rise to viable spore using the CUP-IME1 system https://pubmed.ncbi.nlm.nih.gov/27605516/. As for T2 and NDT80, we know that GAL-NDT80 in combination with GAL4ER does not lead to massive overexpression, and this system has been widely used for studying faithful chromosome segregation during meiosis [1-3]. For T3, we did not manipulate the expression of transcription factors.*

*We have now included a statement in main text:*

*Given that expression of Ime1 and Ndt80 were controlled from heterologous promoters for T1 and T2 in order to obtain a highly synchronous cell population, there is a possibility that this can lead to mis-regulation of a subset of transcripts. However, it’s worth mentioning that the synchronization system has been well characterized previously, and gives rise to viable spores indistinguishable from the wild-type.*

So where does this leave us? The data are valuable but not validated sufficiently for reproducibility and applicability to the quantitative analyses applied later. The chromatin data, both MNASE and comparison in Set mutants, may allow the authors to find qualitative answers about relationships between isoforms that they wish to include without requiring them to over interpret peak values in TSS and TES data. The manuscript would likely improve by focusing on a few strong statements that can be proven.

*We thank the reviewer for the comments. We believe that the new analysis further supports that the TSS-seq data is quantitative and reproducible (see previous points). We are therefore confident that the manuscript in its current form is the best way to go forward.*

Minor points:

1.The authors do not sufficiently recognize the work of Zhang and Neiman. This should be cited in additional places because it first showed that Ndt80 causes expression of transcript isoforms from within genes in gametogenesis.

*We agree that the work of Zhang and Neiman is the first example in yeast meiosis of internal TSS usage. We have now cited the work explicitly.*

2.The title of the manuscript does not fit with the data in it. There is no mention of transcriptional tuning anywhere outside the title and abstract and intro but rather the data shows that there are a variety of relationships including no relationship between isoforms. Tuning means changes in levels that are controlled by a mechanism and the authors do not show any causality in alternative start use and changes in levels of transcription.

*We thank the reviewer for the suggestion. We agree that we have not worked out the mechanism in this work. The initial goal of the work was to identify similar patterns as we identified for NDC80 as described previously [4, 5], as well as to identify new patterns. To prove direct causation, we would have to work out every example as have done for NDC80; genetically manipulate the upstream TSS and examine the effect on main TSS usage. Nevertheless, there are several pieces of evidence that suggest the correlative effects could be causative effects. First, we found that a large subset of transcripts show an inverse correlation; when upstream transcript usage increased the downstream transcript is downregulated. In addition, these correlations directly rely on the expression of transcription factors, and correlate with their binding sites in promoters. Moreover, deleting or depleting chromatin factors (Set2, Set3, FACT) leads to depression at a subset of these loci, indicating that they are required for mediating the repression in the presence of upstream transcription. We agree that we could not prove causation, but the data suggest that this could be the case for many examples. Therefore, we have adjusted the title accordingly (replacing “reveals” by “suggests”).*

*“High-resolution analysis of cell-state transitions in yeast suggests widespread transcriptional tuning by alternative starts”*

3.I did not understand the description of the analyses in the switching section. It seems that the rules described are for identifying instances of replacement of one isoform with another but co-existence and changes in levels could also occur. To measure tuning or switching as the title implies as a goal, one would expect an analysis of temporal patterns not fold changes or expression levels. Why not use these same analyses as were used to globally assess relationships between isoforms? Why does the switching analysis use arbitrary cut offs that require certain fold changes and expression levels? My attempt to understand these categories based on the cited papers in this section did not clarify these choices. Are the authors solely looking for cases that have all the same quantitative features as NDC80 does in these new data?

*We agree that this section is somewhat confusing, therefore we decided to change the section and simplify the analysis. We have now formulated one TSS switching event category. We show all the data points in violin plot (Figure 3c). In short, we have removed different categories of “TSS switching”. Our simplified approach not only allows us to look for cases that have similar quantitative features as NDC80, but also to show that there is a wide variety of possible changes at the main TSS, occurring at the same time when an upstream alternative TSS is upregulated. Our updated violin plots (Fig 3b and 3c) show a large spread in the main TSS log2 fold changes, when an upstream alternative TSS is upregulated, across all 3 transitions. In parallel, we show examples of TSSs with different regulation across time (compare Figures 3e vs 3f). This is a noteworthy point because previously published studies (Cheng et al., 2018) have suggested isoform switching to be widespread in yeast, whereas our TSS resolution data paints a more complex picture.*

4. Some sentences are too vague to be helpful, including "We propose that TSS usage changes drive a widespread dynamic transcriptional program, impacting mRNA and protein production." And "Our data suggest that increased alternative upstream TSS usage can have a range of outcomes on transcription from the main TSS, and upstream long transcript isoforms possess different translation efficiencies."

*We removed that first statement all together. We changed the second statement: “Our data suggest that the transcripts isoforms emanating from upstream alternative TSSs possess different translation efficiencies. “*

5. The discussion ends by saying the data are consistent with previous observations and then simply citing two of the authors recent papers. It would be helpful to discuss how the data fit in the field more broadly.

*We thank the reviewer for the suggestion. We have changed the paragraph and have tried to take examples that put our observations in a broader perspective.*

*“Our data are consistent with previous observations. For example, different degrees of transcriptional repression of canonical transcript isoforms were also observed for a subset of genes expressing LUTIs [6]. Repression of sense mRNA transcription through the act of antisense transcription also has a level dependent effect - the higher the antisense transcription, the stronger the repression of sense transcription [7]. A genome-wide interrogation of single cells of the mouse brain showed that many main and alternative TSS pairs tend to be co-regulated, in particular when closely spaced [8], suggesting conservation from yeast to mammalian cells.*

Reviewer #2:

Review:

This publication by the Van Werven lab aims at describing the changes in TSSs (and TESs) at a genome-wide level during cell-state transitions in S. cerevisiae. To achieve this, the authors use several technics such as TSS-seq, TES-seq, mRNA-seq, TIF-seq and MNase-seq at several key time points during meiosis but also growth in rich medium. The take-home message of the publication is that TSS positioning is highly dynamic during cell-phase transitions and can lead to several outcomes of gene expression. An increase in Upstream Alternative TSSs (UA TSS) leads to either downstream gene repression or activation. This difference appears to mainly be set by the distance between the UA TSS and the original TSS. The authors also describe the importance of chromatin-related factors (Set2, Set3 and Spt16) in the regulation of the original TSS.

This publication is quite amazing in terms of genome-wide data generated and subsequent analyses. However, this might be the weakness of the publication as well. Many different criteria are taken into account for the analyses and this is perhaps why this article lacks a certain homogeneity. Genome Biology appears as a good target for this publication but I would suggest some more experiments, precisions or changes to (try to) improve this work. The comments below should help to make it more accessible to the reader and suitable for publication.

My main comment is that the molecular bases of TSS switching are not directly addressed.

Recent work from Friedman lab (Klein-Brill et al., 2019), Shore and Libri labs (Challal et al., 2018; Kubik et al., 2019) and your lab (Wu et al., 2018) have shown the importance of both transcription factors and nucleosome positioning in TSS regulation.

- Are UA TSS related to new (upstream) or extended NDRs? Does the distance between UA TSS and main TSS influence chromatin structure? MNase-seq data are presented in the supplementary figures only for the internal TSS and main TSS but not for UA TSS. For example, when the distance is <80bp, one might expect that NDRs are not changed whereas a new NDR might appear when the distance is >80bp.

*We thank the reviewer for the comment and suggestion. We have now compared the chromatin structure for genes with an alternative TSS <80bp and >=80bp upstream of the main TSS (Additional file: figure S7a). We indeed see differences between two classes. Genes with an alternative upstream TSS < 80 bp tended to have larger NDR, which likely permissive for expression from multiple TSSs. These genes also showed a defined peak for transcription factor binding. The genes with an upstream alternative TSS >80 bp upstream of the main TSS possessed a smaller NDR and a new NDRs tended to occur around the upstream TSS. As expected, the binding profile for transcription factors is more spread out as they likely control the transcription from the more upstream TSS.*

- The distance between the TATA-Binding Site (TBS) and +1 nucleosome is important for transcription initiation (Kubik et al., 2018). In cases of an UA TSS, is transcription initiation related to another TBS? Same or different TBS for Alternative proximal and Alternative distal classes?

*We thank the reviewer for the suggestion. It is indeed possible that there are multiple TATA binding sequences in the promoters to regulate the upstream alternative TSSs. As suggested, we have examined the TATA box distribution (Additional file 1: figure S7a). While it is clear that there is a good enrichment of TATA binding sequences just upstream of the main TSS, we did not observe a particular difference in patterns between two classes (<80 bp and >=80bp). One obvious explanation is that this is masked by the fact that alternative TSSs are localized at different positions upstream, which will be average out. However, upon close interrogation of the individual plots, we also did not observe clear signals for TATA binding sequences at upstream alternative TSSs. Metagene analysis for TATA box sequence centred on alternative TSSs did not show enrichment (see here below Figure 1). Therefore, we think that alternative TSSs are likely not regulated via canonical TATA binding sequences.*

*Figure 1. Meta-profiles of genes whereby the distance between main and upstream alternative TSS is less than 80 bp (< 80bp) or more than 80 bp (>= 80bp). Signals were centred on the alternative TSS. Displayed are the TATA box motif density (T1, T2 and T3).*

- Are Ume1 and Ndt80 recruited in the vicinity of UA TSSs? This seems true for the internal TSS but not shown for UA TSS.

*We thank the reviewer for pointing this out. We had performed this analysis previously, but this somehow did not make into the final version of the manuscript. We have now included these data (Additional file 1: figure S7a). Indeed, there is evidence that Ume6 and Ndt80 are enriched at promoter sequences of alternative TSS. In particular for the class of transcripts where the distance between main and alternative TSS is greater than 80 bp, we observed distinct peaks for Ume6 and Ndt80 binding.*

Some more specific comments:

- If understood correctly, Figure 2B says that about 3000 genes have Alternative TSS clusters. However, Figure 1F says that around roughly 2000 genes have more than 1 TSS cluster. Where is this discrepancy coming from?

*We thank the reviewer for the comment. In figure 2b the number of alternative TSSs are displayed. Some genes can give multiple alternative TSSs, hence the number is somewhat larger than that in Figure 1f, where the number of genes is displayed. Given that Figure 2 is for understanding the function of alternative TSSs, we prefer to keep the figure as it is.*

- In Figure 2C, the Alt TSS taken into account are the ones that show more than 2 fold increase. So, they are not the same as in Figure 1B for which definition is less strict. Globally, for all the publication, it would be of interest to indicate how many genes are taken into account directly on the graphs (at the top of histograms for example).

*We thank the reviewer for the comment. We agree that it is important to add the number of TSSs/TESs used for the analysis. We attempted to add the numbers to each graph, however, in our opinion it made the graphs quite clogged and less clear. Therefore, we decided to add these numbers to the figure legends. We have now consistently added the numbers used for each violin plot throughout the manuscript in the figure itself or the figure legend.*

- Figure 3C: It seems that relaxed/moderate/strict criteria complexifies the analysis without bringing important novel conclusions.

*We have removed this analysis, and now only show the data for the relaxed cut-off. Instead of a bar graph, we have generated a violin plot and show the separate data point. We think this analysis further supports our conclusions of the manuscript.*

- Figure 3E: It might be good to add a snapshot of an example of TSS switching and TSS co-reg, at least as a supplementary figure.

*We have added IGV screen shots of the TSS-seq data in Additional file: Fig S4c for example genes.*

- Figure 5 is of big interest for the conclusion of the publication. In my experience, trying to find a linear correlation with a good correlation coefficient does not work well (biological responses are not linear most of the time). It is just a suggestion but what about trying to represent the same data with histograms? An example with Figure 5A. Genes might be separated into 2 groups (<80bp and >80bp) and the main TSS fold-change might be represented according to these 2 groups. It will simplify the overall design and will stress what you propose in the model.

*We have adopted the reviewer’s suggestion and have separated genes into two groups (<80 bp and >= 80 bp), while representing main TSS fold change accordingly. The two density plots (new figure 5b) show that at proximal TSS pairs (<80 bp), main TSSs (downstream member of the pair) are biased towards upregulation. We have included the Kolomogorov-Smirnov statistical test to show that the two groups belong to different distributions.*

- Figure 5B: a correlation of -0.1 (even with a good p-value) is not satisfying.

*We agree with the reviewer that -0.1 is poor correlation. We have removed the graph as we do not make significant reference to this figure.*

- Figure 6E: the metagene analysis is presented without statistics; it is therefore difficult to conclude that chromatin structure is really different.

*We agree that statistics would be useful for the analysis of the MNASE-seq data represented in Figure 6e. Given we performed two biological replicates for this experiment, it is tricky to perform statistics on these data. Nevertheless, we were able to calculate how many genes show significant differences in the TSS to -0,5 kb based on DANPOS2. We found that about 30% of depressed genes show significant differences. We have reported this the revised manuscript.*

- Figure 7A: the data are presented with the relaxed criteria. Moderate and strict classes could also be considered. It might increase the statistical power of the analysis.

*We thank the reviewer for the comment. We have removed the different classes of TSS switching from the manuscript as suggested by both reviewers. Given the relaxed TSS switching category contains the strict and moderate genes too, we think it is not likely going to improve the statistics for the further analyses.*

- Figure 7C: some of the cases are not well proven in the publication. For example, I agree that for the distal alternative TSS usage, the case at the bottom is shown in the publication (Strong>Weak>TSS switching> decrease) and well demonstrated. But, for many "protein levels", there is no obvious information in the publication and there should therefore be a question mark. Not sure for example that Proximal>Strong>Weak >co-upreg is really shown in the publication.

*We thank the reviewer for the comment. We agree that there is no clear data for all the classes we propose. Therefore, we have changed the figure and removed the effects on protein expression. We somewhat disagree that there was no demonstration of a Proximal>Strong>Weak>co-upreg effect. We observed a positive correlation for genes with a proximal TSS and relatively low expression of main TSS (Figure 5c). It is worth noting that we do not claim there is causative effect between the proximal TSS and up-regulation of the main TSS. Nevertheless, we think this a valid and important part of the model.*

- There is the possibility that you underestimate the Alternative TSSs isoforms since these transcripts might be discarded by premature termination (Nrd1-Nab3-Sen1 pathway) or NMD. Some isoforms might be transcribed but rapidly degraded giving a low isoform signal while having an impact on the downstream main TSS through chromatin regulation. It is probably really complicated to reveal such a phenomenon but would be nice to discuss it.

*We thank the reviewer for this suggestion. We agree that there is indeed the possibility that we underestimate the number of TSSs. Hence, we added a short section in the discussion.*

*“. It worth noting that we measured steady state levels of TSS usage, which could underestimate the number of TSSs. In particular, the TSSs from noncoding transcripts and transcripts with premature stop-codons or short upstream open reading frames are likely to be discarded rapidly via RNA degradation pathways [9-11].”*

*References:*

*1. Berchowitz LE, Gajadhar AS, van Werven FJ, De Rosa AA, Samoylova ML, Brar GA, Xu Y, Xiao C, Futcher B, Weissman JS, et al: A developmentally regulated translational control pathway establishes the meiotic chromosome segregation pattern. Genes Dev 2013, 27:2147-2163.*

*2. Carlile TM, Amon A: Meiosis I is established through division-specific translational control of a cyclin. Cell 2008, 133:280-291.*

*3. Chia M, van Werven FJ: Temporal Expression of a Master Regulator Drives Synchronous Sporulation in Budding Yeast. G3 (Bethesda) 2016, 6:3553-3560.*

*4. Chen J, Tresenrider A, Chia M, McSwiggen DT, Spedale G, Jorgensen V, Liao H, van Werven FJ, Unal E: Kinetochore inactivation by expression of a repressive mRNA. Elife 2017, 6.*

*5. Chia M, Tresenrider A, Chen J, Spedale G, Jorgensen V, Unal E, van Werven FJ: Transcription of a 5' extended mRNA isoform directs dynamic chromatin changes and interference of a downstream promoter. Elife 2017, 6.*

*6. Tresenrider A, Jorgensen V, Chia M, Liao H, van Werven FJ, Ünal E: Integrated Genomic Analysis Reveals Key Features of Long Undecoded Transcript Isoform (LUTI)-based Gene Repression. bioRxiv.*

*7. Nevers A, Doyen A, Malabat C, Neron B, Kergrohen T, Jacquier A, Badis G: Antisense transcriptional interference mediates condition-specific gene repression in budding yeast. Nucleic Acids Res 2018, 46:6009-6025.*

*8. Karlsson K, Lonnerberg P, Linnarsson S: Alternative TSSs are co-regulated in single cells in the mouse brain. Mol Syst Biol 2017, 13:930.*

*9. Jensen TH, Jacquier A, Libri D: Dealing with pervasive transcription. Mol Cell 2013, 52:473-484.*

*10. Peccarelli M, Kebaara BW: Regulation of natural mRNAs by the nonsense-mediated mRNA decay pathway. Eukaryot Cell 2014, 13:1126-1135.*

*11. Malabat C, Feuerbach F, Ma L, Saveanu C, Jacquier A: Quality control of transcription start site selection by nonsense-mediated-mRNA decay. Elife 2015, 4.*

**Second round of review**

**Reviewer 1**

The revised manuscript from Chia and colleagues is improved from the first submission. However, there remains a major concern that must be addressed for some of the data to be interpretable. The authors argue that TSS sequencing data are linearly quantitative but the evidence provided is not strong and this is critical to interpreting several analyses here. The data in the paper cited from the Winston lab is comparing aggregated data over all genes between two mutants. This would average out differences that may come from library bias for any one transcript and is different than the type of analysis applied in this manuscript. The plots in S1F do not show high enough agreement between the TSS sequencing data and the mRNA sequencing data to justify its use in the quantitative ways implemented in this manuscript.

The authors are correct that this medium agreement is not surprising and it does not reflect a concern with the value of the data. However, it does mean that analyses that show poor or no agreement between sets of TSS sequencing values cannot be interpreted (as in Figure 3) unless additional controls are added to confirm quantitative nature of these data. Observing no signal in this data can either mean no relationship between the transcripts or noise/variability in the data and it cannot be determined which of these is the case for any one instance. Signal that is observed is interpretable, but no signal is not if the noise/variability is high, which S1F suggests. Analysis that compares two sets of noisy/variable values often returns noise/variability and one cannot conclude that this means there is no biological signal.

There is much valuable data in this manuscript that should be published so I would suggest one of two solutions to move forward. Either qRT-PCR or northern blot controls should be added for several cases in which no relationship between TSSs is found to show data that confirm TSS data or it should be stated that cases of no relationship may reflect noise/variability in the data and conclusions should not be made about the biological regulation in those cases.

**Reviewer 2**

The authors have carefully and seriously answered all my comments. The new Figure S7 is of high interest since it takes into account the data of MNase-seq that were a bit tuned-down in the first version of the manuscript. This study provides an amazing amount of data presented in a straightforward fashion so that the main ideas are easily caught by the reader. The revised version of this manuscript should therefore fulfill the requirements for publication in Genome Biology.

**Authors Response**

**Point-by-point responses to the reviewers’ comments:**

Point by point rebuttal:

Reviewer #1:

The revised manuscript from Chia and colleagues is improved from the first submission. However, there remains a major concern that must be addressed for some of the data to be interpretable. The authors argue that TSS sequencing data are linearly quantitative but the evidence provided is not strong and this is critical to interpreting several analyses here. The data in the paper cited from the Winston lab is comparing aggregated data over all genes between two mutants. This would average out differences that may come from library bias for any one transcript and is different than the type of analysis applied in this manuscript. The plots in S1F do not show high enough agreement between the TSS sequencing data and the mRNA sequencing data to justify its use in the quantitative ways implemented in this manuscript.

The authors are correct that this medium agreement is not surprising and it does not reflect a concern with the value of the data. However, it does mean that analyses that show poor or no agreement between sets of TSS sequencing values cannot be interpreted (as in Figure 3) unless additional controls are added to confirm quantitative nature of these data. Observing no signal in this data can either mean no relationship between the transcripts or noise/variability in the data and it cannot be determined which of these is the case for any one instance. Signal that is observed is interpretable, but no signal is not if the noise/variability is high, which S1F suggests. Analysis that compares two sets of noisy/variable values often returns noise/variability and one cannot conclude that this means there is no biological signal.

There is much valuable data in this manuscript that should be published so I would suggest one of two solutions to move forward. Either qRT-PCR or northern blot controls should be added for several cases in which no relationship between TSSs is found to show data that confirm TSS data or it should be stated that cases of no relationship may reflect noise/variability in the data and conclusions should not be made about the biological regulation in those cases.

*We thank the reviewer for these valuable comments and suggestions. We agree that ideally further experimentation should be performed to determine whether the cases that show no relationship between TSSs, are due to technical noise. We also agree that the agreement between mRNA-seq and TSS-seq is less ideal. A look at other datasets in the literature suggests that it may be challenging to improve this further. However, it is worth noting that the correlation between biological repeats in TSS-seq data is very good (typically R=0.95 or higher), suggesting the signals we measured by TSS-seq are reproducible and are perhaps not that noisy.*

*Quantitative RT-PCR is not able to discriminate between overlapping mRNA isoforms, hence northern blot would be the only appropriate technique to confirm the cases that do not display a relationship. We have performed northern blots for some cases, but most of these were on examples that showed good correlation between alternative and main TSS usage. For one example we have performed a northern blot, ORC3, where we did not observe a correlative change between the upstream alternative and main TSS in our TSS-seq data. We included the ORC3 data here (Figure 1 of the rebuttal document). Preliminary analysis suggests that ORC3 main transcript does not change as detected by northern blot when the longer form is induced during transition 2. The analysis by northern blot is not complete hence we cannot include this in the current manuscript. However, we also note similar results with northern blot analysis of ORC3 in a sporulation time course in Cheng et al., 2018, figure 5A, bottom panel. Their analysis also showed that levels of the main ORC3 isoform (canon.) remained quite stable despite strong induction of the longer isoform (LUTI).*

*Figure 1. The expression of ORC3 isoforms during T1 and T2 detected by northern blot and TSS-seq.*

*Figure 5A from Cheng et al. 2018*

*Performing additional experiments with northern blots for other genes is currently not possible due to fact that first authors have left the laboratory. Therefore, we have reformulated our conclusions and have discussed the possibility that noise variation should be considered when interpretation some of the data as suggested by the reviewers comment. I have outlined which sections of the manuscript have been altered.*

*In the Results page 8 we added:*

*Additionally, the three independent biological replicates in this study highly correlated with each other (Additional file 2). The TSS-seq and TES-seq datasets correlated well with the RNA-seq dataset (TSS-seq vs RNA-seq and TES-seq vs RNA-seq) (Fig. 1h and Additional file 1: Fig. S1f, S1g and Additional file 3, 4 and 5). However, it is worth noting that genes with relatively low expression levels for the RNA-seq correlated less well with TSS-seq and TES-seq, which could be due to noise in the data. Nevertheless, these data indicate that our TSS-seq and TES-seq datasets can be largely used for quantitative estimates of steady state levels of TSS and TES usage.*

*And page 15:*

*As a caveat, we cannot rule out the impact of noise in the TSS-seq data, for the examples in which we observed little to no change in main TSS signals in the presence of increased upstream alternative TSS expression. Nevertheless, our analysis indicates that increased expression from upstream alternative TSSs is linked to various outcomes on the expression of the matching main TSSs and is not always associated with gene repression.*

*In the Discussion page 27 we added:*

*Although our TSS-seq data showed good reproducibility over three biological repeats (Additional file 2), we cannot rule out the role of experimental noise in why we observed co-existence of main and alternative TSSs for some gene loci. Further functional analysis and the use alternative techniques such as fluorescent in-situ hybridization (FISH) or northern blot will be essential for studying the relationships between main and alternative TSSs more closely.*

Reviewer #2:

The authors have carefully and seriously answered all my comments. The new Figure S7 is of high interest since it takes into account the data of MNase-seq that were a bit tuned-down in the first version of the manuscript. This study provides an amazing amount of data presented in a straightforward fashion so that the main ideas are easily caught by the reader. The revised version of this manuscript should therefore fulfill the requirements for publication in Genome Biology.

*We thank the reviewer for the positive feedback on the revised manuscript.*

**Third round of review**

**Reviewer 1**

In order to more appropriately address our major concern of the possible over-interpretation of data that may not be quantitative, the statement on page 13 should say "This suggests that either most LUTI regulated genes do not switch expression from protein coding TSS to the LUTI TSS or that noise in the TSS-seq data prevents detection of switching" which more accurately represents the results than the current statements.
